# Evaluating a large-scale online behaviour change intervention aimed at wildlife product consumers in Singapore

**DOI:** 10.1371/journal.pone.0248144

**Published:** 2021-03-24

**Authors:** Hunter Doughty, E. J. Milner-Gulland, Janice Ser Huay Lee, Kathryn Oliver, L. Roman Carrasco, Diogo Veríssimo

**Affiliations:** 1 Department of Zoology, University of Oxford, Oxford, United Kingdom; 2 Oxford Martin Programme On The Illegal Wildlife Trade, University of Oxford, Oxford, United Kingdom; 3 Asian School of the Environment, Nanyang Technological University of Singapore, Singapore, Singapore; 4 Faculty of Public Health and Policy, London School of Hygiene and Tropical Medicine, London, United Kingdom; 5 Department of Biological Science, National University of Singapore, Singapore, Singapore; 6 Institute for Conservation Research, San Diego Zoo, Escondido, CA, United States of America; Qinghai University, CHINA

## Abstract

Interventions to shift the behaviour of consumers using unsustainable wildlife products are key to threatened species conservation. Whether these interventions are effective is largely unknown due to a dearth of detailed evaluations. We previously conducted a country-level online behaviour change intervention targeting consumers of the Critically Endangered saiga antelope (*Saiga tatarica*) horn in Singapore. To evaluate intervention impact, we carried out in-person consumer surveys with >2,000 individuals pre- and post-intervention (2017 and 2019), and 93 in-person post-intervention surveys with traditional Chinese medicine (TCM) shopkeepers (2019). The proportion of self-reported high-usage saiga horn consumers in the target audience (Chinese Singaporean women aged 35–59) did not change significantly from pre- to post-intervention (24.4% versus 22.6%). However, post-intervention the target audience was significantly more likely than the non-target audience to accurately recall the intervention message and to report a decrease in saiga horn usage (4% versus 1% reported a behaviour change). Within the target audience, high-usage consumers were significantly more likely than lower-usage consumers to recall the message and report a behaviour change. Across respondents who reported a decrease in saiga horn usage, they cited the intervention message as a specific reason for their behaviour change significantly more than other reasons. Additionally, across all respondents, the belief that saiga is a common species in the wild decreased significantly from pre- to post-intervention. TCM shopkeepers, however, cited factors such as price and availability as the strongest influences on saiga horn sales. In sum, the intervention did significantly influence some consumers but the reduction of high-usage consumer frequency was not significant at the population level. We explore reasons for these findings, including competing consumer influences, characteristics of the intervention, and evaluation timing. This work suggests our intervention approach has potential, and exemplifies a multi-pronged in-person evaluation of an online wildlife trade consumer intervention.

## Introduction

A multitude of biodiversity conservation efforts are conducted each year to address unsustainable or illegal wildlife trade [1]. At the consumer end of trade chains, these efforts are predominately in the form of interventions targeting individual consumer behaviour [2], as opposed to, for example, those targeting suppliers. At minimum 2.35 billion US dollars were spent between 2010 and 2018 to address illegal wildlife trade, though only 104 million were used for communications and awareness, including consumer-focused projects [3]. Despite this overall investment, illegal or unsustainable wildlife trade continues to play an important role in the loss of wildlife globally [4]. The limited evidence base underpinning many consumer demand reduction interventions, and the lack of detailed evaluation of intervention impacts, have thus been critiqued [5, 6], which follows similar calls for empirical intervention evaluation across conservation sciences [7, 8].

Applied behavioural sciences in fields like public health and international development offer examples of intervention evaluation approaches and standards that could be useful to conservation and sustainability projects [9, 10]. For example, evaluations of interventions in complex socio-political contexts or of those that target sensitive behaviours (e.g. HIV prevention among girls in Mozambique, and reducing intimate partner violence across Europe [11, 12]) provide real-world examples of challenges that often characterise conservation study contexts. Such applied behavioural science evaluations strive to assess the causal effect of an intervention and rule out alternative explanations for observed effects [8]. Some argue these evaluations should also assess how that effect occurred [13]. Literature across the applied behavioural sciences include a plethora of methods available for conducting evaluations to meet these aims. For example, researchers can assess temporal changes in a target audience’s behaviour through longitudinal data, in which the same individuals are measured at various time points, or through repeated cross-sectional data, in which a different representative sample of the population is measured at each time point [14]. When the use of treatment-control designs is not feasible or appropriate [15, 16], other evaluation approaches include using multiple data sources in conjunction: e.g. combining self-reported surveys and third-party measurements [17]. These multi-pronged approaches are also helpful in providing a more complete picture of intervention effects, and reducing the likelihood that evaluations are biased by the limitations of any one method.

The type of data collected also varies across evaluations, and can dictate what the evaluation is able to show. Self-reported data are directly collected from respondents (e.g. consumer surveys) and are commonly used in wildlife trade evaluations [2]. These can be useful for many reasons, such as when the behaviour would be otherwise difficult to measure. However, self-reporting is inherently open to response biases [18]. Third-party reported data come from sources other than the person of interest, such as a building’s electricity usage meter [19], or key-informant interviews with sellers of wildlife products. These are not affected by self-reporting biases but have their own limitations, such as being access-limited. In addition, mixed-method approaches employing both quantitative and qualitative data can provide evaluations that are both in-depth and statistically robust [20]. Mixed-method approaches are also useful in creating funnels of attribution [13], which visually detail steps along a causal chain leading individuals from the target behaviour, through intervention exposure, and subsequent behaviour change. Funnels of attribution help elucidate causal mechanisms behind intervention impact and any impact heterogeneity (i.e. variability) by showing points along the causal chain where the number of individuals decreases, which could, for example, indicate unaccounted-for barriers in the behaviour change process. Regardless of the chosen evaluation approach, arguably a comprehensive evaluation aims to use robust and feasible means to assess who in the target audience the intervention worked for and did not work for, and why [13].

### Study system

The saiga antelope (*Saiga tatarica*) is a Critically Endangered species from Central Asia [21], whose horn is used in traditional Chinese medicine (TCM) to treat fever and “heatiness*”* (a TCM state of illness with symptoms like cough and sore throat). Poaching for this horn is a major threat to saiga survival [21]. Singapore is recognized as a top saiga horn consumer country [22], and within the country, saiga horn (marketed as líng yáng, 羚羊) is legal and commonly available [23]. In February–April, 2019, the authors implemented a country-level online behaviour change intervention targeting Chinese Singaporean women, aged 35–59 years old, to reduce saiga horn usage in Singapore (see [24]). This target audience was chosen because baseline research found middle-aged women were among those most likely to use saiga horn themselves and were the most likely group to purchase saiga horn for other people [23].

Using baseline research [23, 25], human behaviour theory and prior studies [25], as well as piloting [24], an intervention design was chosen: using targeted online advertisements to strategically promote several online news articles in Singapore that discussed information about saiga horn (published as [24]). This intervention differed from traditional mass or social media campaigns that usually promote their own original content, by instead promoting third-party news articles published in established media outlets. This strategy allowed us to harness the influence of health news stories [26] while still making the most of targeted advertising’s ability to segment and reach consumers with specific messaging [27]. The influences of health news [26], online repeat message exposure [28] and online social reinforcement [29] employed in this intervention, allowed us to extensively spread this news content throughout our target audience’s social network. Pilot focus groups with the target audience suggested that socially-framed information on the saiga’s conservation status, delivered by Singapore-based messengers, had a strong impact on the target audience’s behavioural intention to use saiga horn [24]. Thus, the intervention message (included in the news articles and advertisements) presented the source of saiga horn products in a way that framed users as unwitting consumers of a threatened wildlife species. It also implied that these consumers, based on socially accepted perceptions in Singapore, would want to know the impacts of their consumption [24].

An initial online analysis of audience engagement showed overwhelmingly desirable audience engagement: 63% of audience-created Facebook content was identified as positive towards the intervention message, while only 13% was identified as negative [24]. In order to robustly determine the intervention’s impact on the target audience, however, we conducted a multi-pronged evaluation–which is the focus of this manuscript. This evaluation consisted of thorough in-person surveys with both consumers and TCM shopkeepers, to determine whether the following hypothesized stages of impact occurred:

- the target audience, and high-fidelity saiga horn consumers within this group (i.e. those who consider saiga horn a product they use most often), remembered the intervention message more than the non-target audience, and decreased their saiga horn consumption more than the non-target audience;
- TCM shopkeepers remembered the intervention message and subsequently discerned changes in saiga horn sales among the target audience more than the non-target audience.

This work offers a rare example of both a detailed multi-pronged pre-post evaluation of a large-scale wildlife trade intervention, and an assessment of impacts from an online intervention on offline behaviour.

## Methods

**Fig 1:**
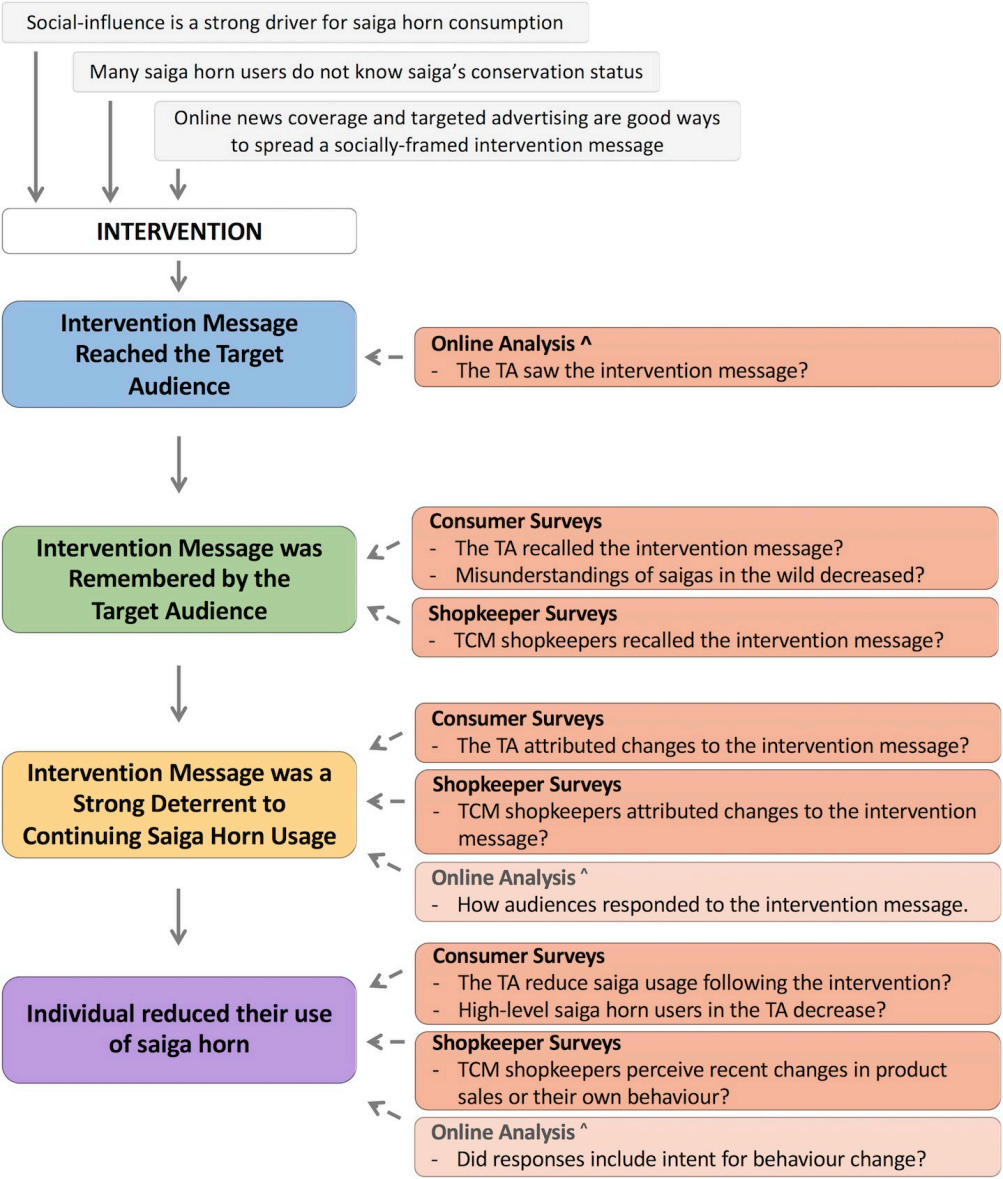
Evaluation theory-of-change, showing the dataset(s) used to assess assumed stages (blue, green, yellow, and purple boxes) resulting from the intervention. The top grey-boxed stages include assumptions underpinning the intervention and the intervention design. ^ See [24] for those initial stages and online analysis findings. The final two stages (yellow and purple) were assessed primarily using consumer and shopkeeper surveys, supported by the online analysis (indicated by transparent colouring). TA = target audience (middle-aged Chinese Singaporean women).

Given the nature of our intervention [24] we were not able to constrain the intervention to a subgroup within our target audience, thus a standard treatment-control design was not feasible [15]. An initial online analysis was previously conducted using advert performance data provided by the advertising platforms and Facebook user-created content (see [24]). This analysis assessed whether the intervention message reached the target audience and provided a preliminary assessment of audience response and engagement (Fig 1). In order to assess all assumed stages of impact resulting from the intervention (Fig 1), we then carried out the full evaluation in this manuscript. Our in-person evaluation included both repeated cross-sectional intercept surveys with the consumer population (pre- and post-intervention, in 2017 and 2019), as well as a cross-sectional survey of TCM shopkeepers (post-intervention, in 2019).

### Intervention message recall

#### Consumer survey

In the post-intervention survey, to measure recall of the intervention message among consumers without prompting them on the content of that message, we stated to respondents only that there had been some ‘media attention in the last four months about a TCM treatment for heatiness and fever called líng yáng’ (see S2 File for the full survey). We then asked respondents if they had heard about it. All those who indicated that they had, or may have, heard about this media attention were labelled as stating intervention message recall. We then asked them how they heard about it, and what the media attention was about. The latter was a free-response question. The answers were coded for content by HD and verified by DV. Answer content relating to the saiga as a threatened or protected animal, the poaching of saiga, and raising awareness of saiga conservation, were taken to indicate that the respondent was accurately recalling elements of the intervention message, and such respondents were labelled as having ‘accurate intervention recall’. Responses could contain more than one type of content.

In both the pre- and post-intervention surveys, respondents were also asked which animals they thought were common in the wild (out of a list of animals often used in TCM), in order to assess respondents’ awareness of the saiga’s threatened status. We chose to ask about ‘commonness’ rather than ‘rarity’ because we felt it provided a more conservative assessment of consumers perception of threatened species. This question was asked prior to any questions regarding the intervention in the post-intervention survey.

#### Shopkeeper survey

For the post-intervention shopkeeper survey, we found during piloting that an introduction was needed to justify to shopkeepers why we were bringing up media attention around saiga horn without prompting them to our research interests. Thus, our researcher stated that there were many factors that can influence or change customers’ choices, and that to understand the role of media better we were going to ask about recent media attention from the past four months around saiga horn (see S2 File for the full survey). We then asked the shopkeepers if they recalled this media attention and how they were exposed to it. The recall answers were coded similarly to the equivalent questions in the consumer survey. Prior to these intervention specific questions, like in the consumer survey, we asked shopkeepers which animals used in TCM were common in the wild. We recorded both the specific animals mentioned and any additional information around why they answered as they did.

### Behaviour change and reasons for that change

#### Consumer survey

There were three means of assessing self-reported behaviour change among the target audience. Firstly, we compared the proportion of ‘high-fidelity’ saiga horn users in the pre- to post-intervention datasets. Since the target audience for the intervention was a demographic group, it included both saiga horn users and non-users. We characterised ‘high-fidelity’ saiga horn users within this group as those who selected saiga horn as a fever/heatiness treatment they use *most often* during questions about their overall treatment preferences. We chose to focus on high-fidelity users in the pre-intervention survey [23] because impacting their consumption in an intervention would yield the greatest per-person reduction in saiga horn usage. To minimise cognitive burden in the survey [30], we also had to balance how many questions we could ask about level of saiga use with the other questions of interest. As such, our post-intervention survey only measured high-fidelity saiga horn usage in the treatment preference questions, so that the measure of saiga horn usage was consistent between pre- and post-intervention surveys. We realise the limitations that this choice entails, in that we were unable to assess temporal changes in lower-fidelity saiga horn users.

Secondly, in the post-intervention survey, immediately after respondents discussed their fever/heatiness treatment preferences, they were asked whether their preferences had changed for any reason in the last four months (without mention of the intervention). Thirdly, in the post-intervention survey, respondents who stated that they recalled the ‘recent media attention’ about saiga horn were asked whether this media attention affected their choice to use saiga horn. At this point, respondents indicated specifically whether prior to the media attention they used saiga horn and whether they currently use it. This was followed by an open-ended ‘why’ question (S2 File). Through these intervention-specific answers we identified: pre-intervention saiga horn users of any fidelity level, whether these users decreased their usage post-intervention, and their reason for changing behaviour.

#### Shopkeeper survey

Like the consumer surveys, the first questions assessing behaviour change were open-ended and were asked after shopkeepers described their customers’ heatiness/fever treatment preferences. Later in the survey, shopkeepers were asked whether, in their opinion, the recent media attention had changed sales of saiga horn to their middle-aged female customers, or their overall customer base. We then asked why and to what extent sales had changed. Additionally, we asked whether they thought sales of alternative products had changed, and whether they themselves had changed what products they recommended to customers.

### General survey details

#### Consumer survey

The consumer surveys were conducted pre- and post-intervention (in 2017 and 2019). These surveys were overall similar with the main exception being that behaviour change and intervention-specific questions were only asked in the post-intervention survey. So that our pre- and post-intervention surveys would be comparable, the post-intervention surveys employed the same sampling method and locations, general survey format and question phrasing, and general data analysis methods, that were used in the pre-intervention surveys (i.e. [23]). In any case, it was important to assess the newly added questions, so the post-intervention survey was piloted at the National University of Singapore and a public mall not used in our study (n = 50 respondents). Both pre and post-intervention surveys involved university-aged Chinese Singaporean students (fluent in both English and Mandarin) conducting in-person, tablet-based surveys with Chinese Singaporean members of the public, outside of public places such as malls and food centres. These locations were selected to capture differing socio-demographic levels, and survey collection timings were stratified across day of week and time of day. Saiga horn is non-sensitive to consumers and widely sold [23], so sensitive questioning techniques were not necessary. See S1 File and [23] for further methodology details, and S2 File for the full survey.

We designed our survey so as to reduce social desirability bias (i.e. unintentionally prompting respondents to answer a certain way based on their perceptions of the researchers’ intentions/perceptions [18]). We did this particularly through neutral question phrasing (e.g. by asking which products consumers use, with saiga horn as just one answer option, rather than asking respondents directly if they use saiga horn). Additionally, we chose to survey two months after the intervention ended in order to trade-off two opposing biases: 1) if the recall period is too long, respondents may inaccurately recall their own behaviour [18] or mis-attribute their behaviour to other media articles coming out in the interim; and 2) behaviour change often decays with time [31], so allowing more time to pass may give a more realistic assessment of medium or long term intervention effects.

All statistical analyses for the consumer survey results were conducted using the R statistical environment [32]. For pre- and post-intervention comparisons we used the MatchIt package to match the 2017 and 2019 datasets, as an added safeguard against possible undetected sampling variability between years [33]. Specifically, we performed an ‘optimal’ match, with a ‘Mahalanobis’ distance measure, and a 1:1 ratio. Variables matched include Chinese dialect, education, generation Singaporean, religion, and target audience (for the total sample comparisons). We then used Generalized Linear Models (GLMs) with sum contrasts applied [34], to assess differences (e.g. saiga horn usage) between: pre- and post-intervention datasets, post-intervention target audience and other respondents, and post-intervention high-fidelity users and other respondents within the target audience (Table 1). We chose GLMs because they account for differences between the datasets, as well as variance caused by other demographic factors when comparing across groups. We next used two-sample z-tests for equality of proportions with a continuity correction to assess intervention message exposure sources, and reasons for behaviour change. This test was chosen because the respondent’s answers on these questions were not mutually exclusive. See S1 File for full statistical analyses methods.

**Table 1:** Consumer survey analysis questions answered using statistical tests.

**GLMs with sum contrasts applied**
**Question asked**
Dependent = Independent variables
“+” indicates the addition of a stand-alone variable
“–” indicates the interaction of two variables and their main effects
**Did high-fidelity saiga horn use, across the total samples, change from 2017 to 2019?**
High-fidelity use = year–target audience + Chinese dialect + education + generation Singaporean + religion
**Did high-fidelity saiga horn use, in the target audience, change from 2017 to 2019?**
High-fidelity use = year + Chinese dialect + education + generation Singaporean + religion
**Did misconceptions of saigas being common in the wild, across the total samples, change from 2017 to 2019?**
Misconception = year–target audience + Chinese dialect + education + generation Singaporean + religion
**Did misconceptions of saigas being common in the wild, in the target audience, change from 2017 to 2019?**
Misconception = year + Chinese dialect + education + generation Singaporean + religion
**Did the 2019 target audience accurately recall the intervention more than the non-target audience?**
Accurate recall = target audience + Chinese dialect + education + generation Singaporean + religion + income
**Did 2019 high-fidelity users in the target audience accurately recall the intervention more than others?**
Accurate recall = high-fidelity user + Chinese dialect + education + generation Singaporean + religion + income
**Did the 2019 target audience change their behaviour more than the non-target audience?**
Decrease usage = target audience + Chinese dialect + education + generation Singaporean + religion + income
**Did 2019 high-fidelity users in the target audience change their behaviour more than others?**
Decrease usage = high-fidelity user + Chinese dialect + education + generation Singaporean + religion + income
**Two-sample Z-tests**
**Was one (or more) intervention exposure source reported more often than other sources?**
Proportion of one exposure source is the same or different from proportion of second exposure source
**Was one (or more) reason for behaviour change reported more often than other reasons?**
Proportion of one reason is the same or different from proportion of second reason

GLM variable descriptions and variable levels are detailed in S1 File. Additional independent variables aside from the variable of interest (e.g. year or target audience depending on the question) were included to give a more conservative assessment of possible associations between the dependent variable and the independent variable of interest. All demographic variables were also described in detail in the baseline survey [23]. Target audience = women aged 35–59 years old. 2017 = pre-intervention sample; 2019 = post-intervention sample.

#### Shopkeeper survey

The TCM shopkeeper survey was conducted post-intervention in 2019. We defined anyone working at the counter during a survey as a shopkeeper. In the pre-intervention consumer survey, TCM shopkeepers were a top recommender of saiga horn, and TCM stores were the most commonly cited location to purchase saiga horn [23]. Thus, TCM shopkeepers were well-placed to witness potential changes in saiga horn sales trends.

One researcher from the same team who conducted the consumer surveys, surveyed TCM shops within the Planning Areas (i.e. neighbourhood districts) or surrounding Planning Areas where we conducted our consumer surveys–in order to have some correspondence between consumer and shopkeeper surveys [35] (S1 File). An online search for ‘TCM stores’ using Search.insing.com yielded about 900 results across Singapore. With this number as an estimated total for TCM shops, we aimed to survey 90 shops. This number would give us estimates with a 10% margin of error and within a 95% confidence level (as determined by a sample size calculation; assuming random sampling) which we felt was acceptably robust and feasible to obtain [36]. Using this directory and Google Maps to identify shops, we randomly selected shops to visit using a random number generator. When shops did not sell saiga horn-like products (e.g. acupuncturists’ offices), or were permanently closed, we randomly selected another shop. Those that were open and did sell relevant products (i.e. TCM products similar to saiga), were asked to take the survey. Regardless of whether they agreed to take the survey, we used visual cues and discussions with the shopkeeper to assess whether they sold saiga products. Visible evidence included: products that were labelled as ‘líng yáng’ with no reference to them being made from a non-saiga alternative; products that explicitly stated they included saiga horn; and products that were evidently saiga horn (e.g. intact horns). The survey was piloted with 20 TCM shops not included in our study. This piloting process, along with iterative survey drafts, helped to minimise social desirability bias and ensure shopkeepers felt comfortable discussing sales trends and customer preferences. See S1 File for additional methodological details and S2 File for the full survey.

### Ethics

This research was approved by the Oxford Internet Institute’s Departmental Research Ethics Committee, University of Oxford (SSH-OII-C1A-19-005), and the Institutional Review Board, Nanyang Technological University (IRB-2017-04-018-01).

## Results

**Fig 2:**
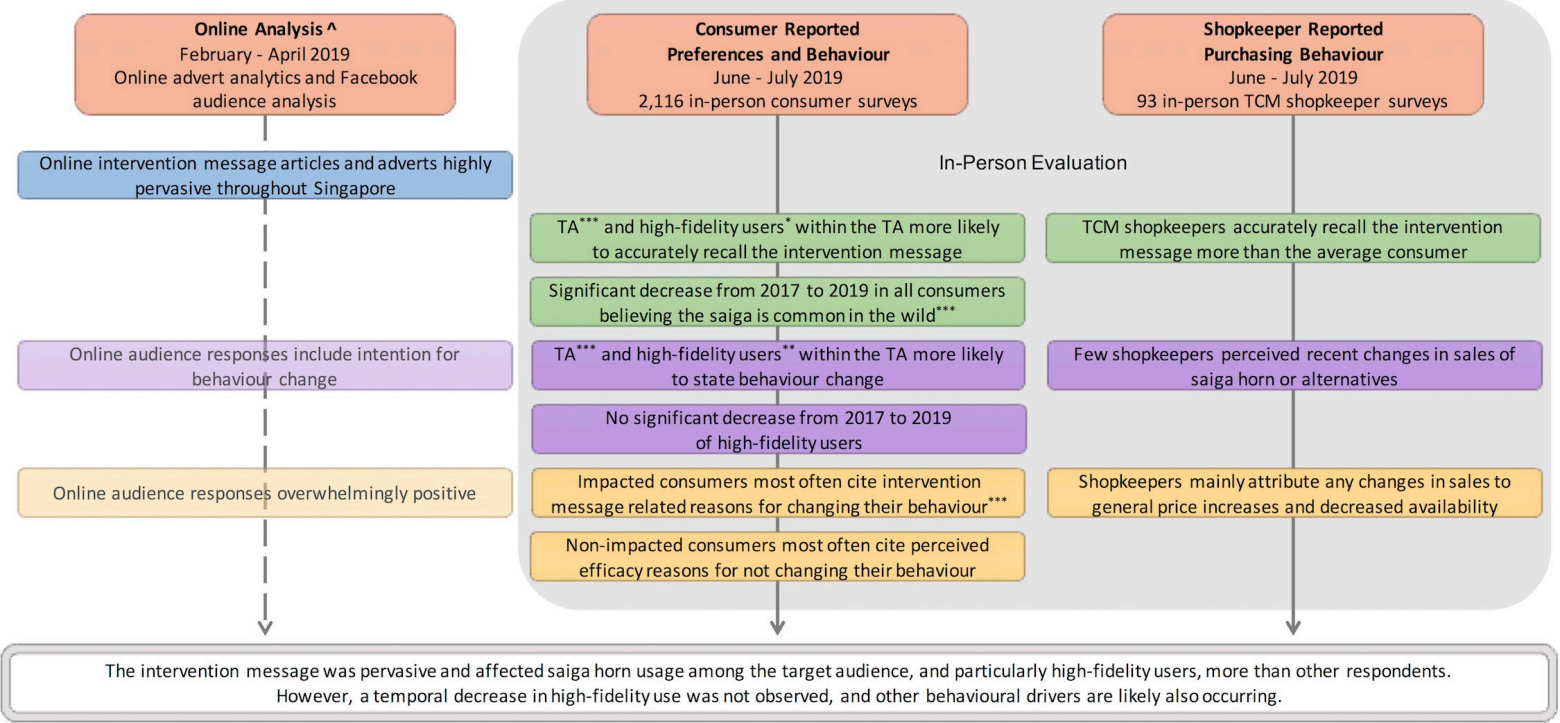
Evaluation summary, including key results from multiple datasets that most elucidated intervention effects. Result box colours correspond to the Theory of Change stages outlined in Fig 1. Transparent colouring indicates a result was supporting but not central to assessing a stage. TA = target audience (middle-aged Chinese Singaporean women). Statistical significance denoted with * for p-value < 0.05, ** for p-value < 0.01, and *** for p-value < 0.001. ^ Online performance findings are presented in [23].

An evaluation summary in Fig 2 highlights the key results which show intervention effects along the stages of intervention impact from our Theory of Change in Fig 1. The full results and statistical findings behind this summary are discussed in the following sections. A total of 2,116 consumer respondents were surveyed post-intervention in June-July 2019 (see S3 File for raw data). For pre-post intervention comparisons, this dataset was compared against the 2,294 consumer respondents surveyed pre-intervention in June-July 2017 [23]. TCM shopkeepers at 119 shops were asked to take the survey. Out of which, 93 shopkeepers accepted (see S3 File for raw data). All 119 shops were assessed for the presence of saiga horn: 104 had visible saiga horn-like products, and 64 shopkeepers verbally indicated they sold saiga horn during survey-related discussions. We thus confirmed the likely presence of saiga horn at all surveyed shops. There were 116 non-relevant or permanently closed shops that were visited but not surveyed.

### Intervention message reach

#### Online analysis

Detailed in Doughty et al. 2020, the articles discussing the intervention message were published in English and Chinese on at least seven news outlets online, many of which are widely read in Singapore. Our online advertisements promoting these articles ran through Facebook, Google, and Outbrain, and were shown to consumers almost 5 million times. Through Facebook alone, these advertisements reached 479,258 women in Singapore aged 35–59 [24].

### Intervention message recall

#### Consumer survey

**Fig 3:**
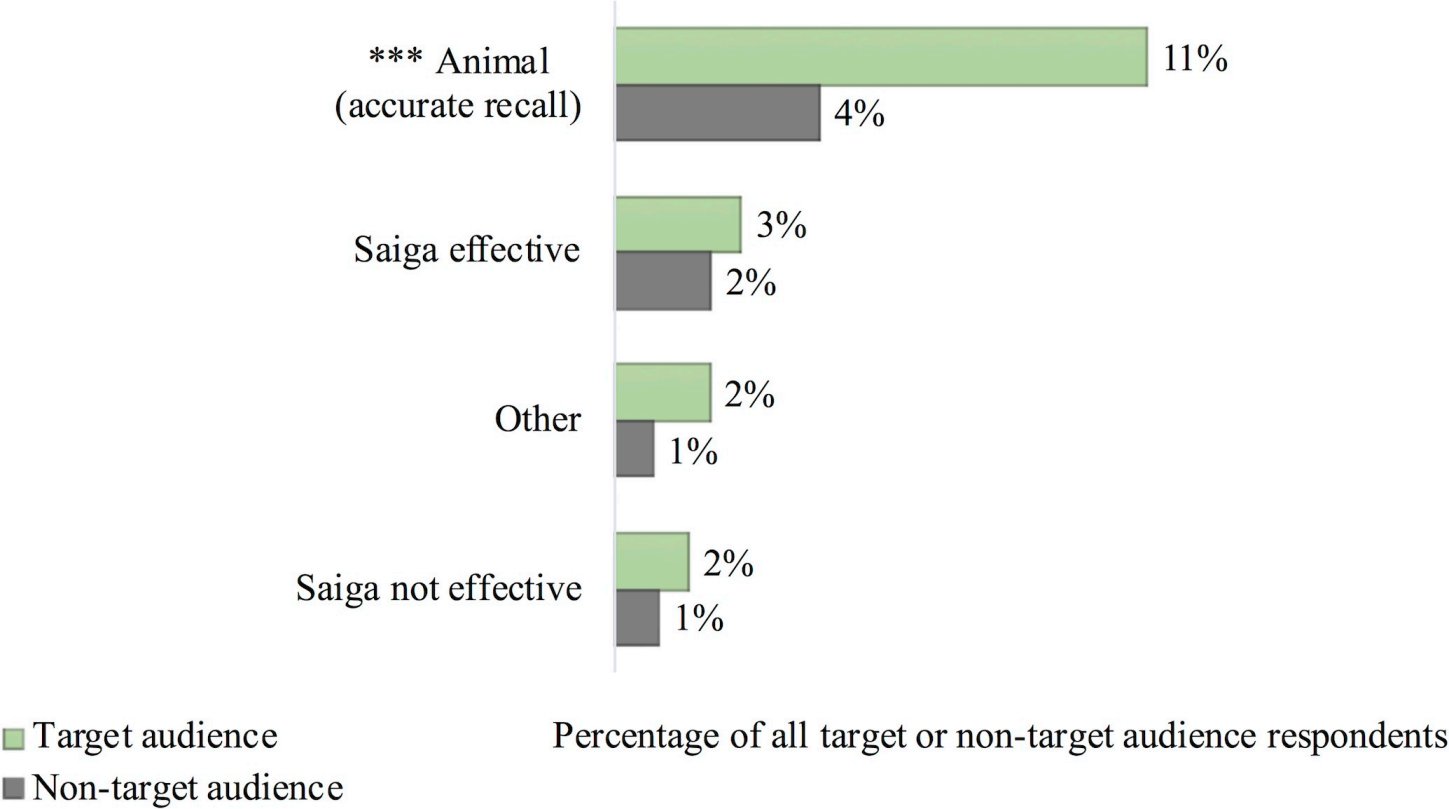
Content of the ‘media attention’ respondents described. ‘Animal’ content refers to generally accurate descriptions of the intervention message. Percentages are out of the total number of target or non-target audience respondents (438 and 1,678 people respectively). *** denotes a p-value <0.001 significant association between the target audience and accurate intervention recall.

Overall, 195 respondents post-intervention stated they recalled some recent media attention about saiga horn (including 69 target audience respondents). Based on descriptions given by respondents, 124 people (6%) accurately recalled the intervention message (including 50 target audience respondents; Fig 3). Inaccurate descriptions of the intervention message were most often about saiga horn’s effectiveness/non-effectiveness (S4 File).

Target audience respondents were significantly more likely than other respondents to accurately recall the intervention message (ß = 0.94, z = 4.69, p = 2.8x10^-6^; Fig 3). Within the target audience, high-fidelity users were significantly more likely than other respondents to accurately recall the intervention message (ß = 0.46, z = 2.02, p = 0.043). See S4 File for full consumer survey GLM outputs along with additional findings on consumer treatment preference trends.

The most common ways any respondent heard about the intervention message were the news, followed by social media (S4 File). Respondents with accurate intervention recall were significantly more likely to cite the news or social media over other sources (χ^2^(1, *N* = 124) = 15.81, *p* = 3.5x10^-5^; χ^2^(1, *N* = 124) = 14.88, *p* = 5.7x10^-5^ respectively). Respondents with inaccurate intervention recall were significantly more likely to cite family and friends (χ^2^(1, *N* = 90) = 3.74, *p* = 0.027; χ^2^(1, *N* = 90) = 3.78, *p* = 0.026 respectively).

Regarding respondents’ perception of wild saiga population commonness: for both the total sample, and the target audience specifically, pre-post intervention comparisons show that respondents in the post-intervention survey were significantly less likely than those in the pre-intervention survey to believe saiga antelopes are common in the wild (ß = -0.44, z = -5.22, p = 1.8x10^-7^; and ß = -0.36, z = -2.25, p = 0.024 respectively) (S4 File). Proportions decreased from to 28% to 21% across the total sample, and from 32% to 26% in the target audience.

#### Shopkeeper survey

A total of 23 shopkeepers stated they recalled recent media attention about saiga horn. Eight shopkeepers (9%) accurately recalled the intervention message. Inaccurate descriptions of the intervention message were most often about saiga being effective, ‘other’ answers, and saiga bans/restrictions (S5 File). Shopkeepers predominately stated they heard about this recent media attention through ‘news outlet/newspaper’ (n = 13) and ‘other’ sources (n = 8) (S5 File). When asked to elaborate on ‘other’, shopkeepers mentioned things like company training books, personal experience, Google searches, and radio broadcasts. Those with accurate intervention recall specifically listed these sources and social media the most often.

When asked if species used in TCM were common in the wild, 16% of shopkeepers stated saiga was common in the wild. Sea cucumber (45%) and goat (34%) were the species most perceived as common in the wild, while rhino (1%) and turtle (0%) were the least. When answering this question the shopkeepers discussed: saiga being a protected/threatened species (n = 16), other species populations doing well (n = 16), restrictions on saiga products (n = 14), and farming of other species (n = 13) (S5 File).

### Behaviour change and reasons for that change

#### Consumer survey

High-fidelity use did not differ significantly from pre- to post-intervention (Table 2). During general preference questions in the post-intervention survey, no respondent who stated there had been a recent change in their herbal or biomedical treatment preferences mentioned they had switched to these options from saiga horn, or referenced the intervention. However, 9 people (3 target audience respondents) who had selected saiga horn as a product they purchase ‘most often’ (and thus were categorised as high-fidelity users), also stated that their preference had changed away from saiga horn in the last few months (S4 File). Four of them (1 target audience respondent) discussed herbal alternatives they were switching to. Two respondents (1 target audience respondent) specifically stated the change was due to news/Facebook posts.

**Table 2:** Comparing high-fidelity saiga horn user frequencies pre- and post-intervention. The proportion of high-fidelity users in the total sample, and the target audience specifically, are shown.

	Pre (2017)	Post (2019)
**Total sample:**	2,294 respondents	2,116 respondents
High-fidelity users	438 (19%)	389 (18%)
—Those who use saiga horn themselves	403 (18%)	343 (16%)
—Those who buy saiga horn for others	223 (10%)	228 (11%)
**Target audience:**	447 respondents	438 respondents
High-fidelity users	109 (24.4%)	99 (22.6%)

**Fig 4:**
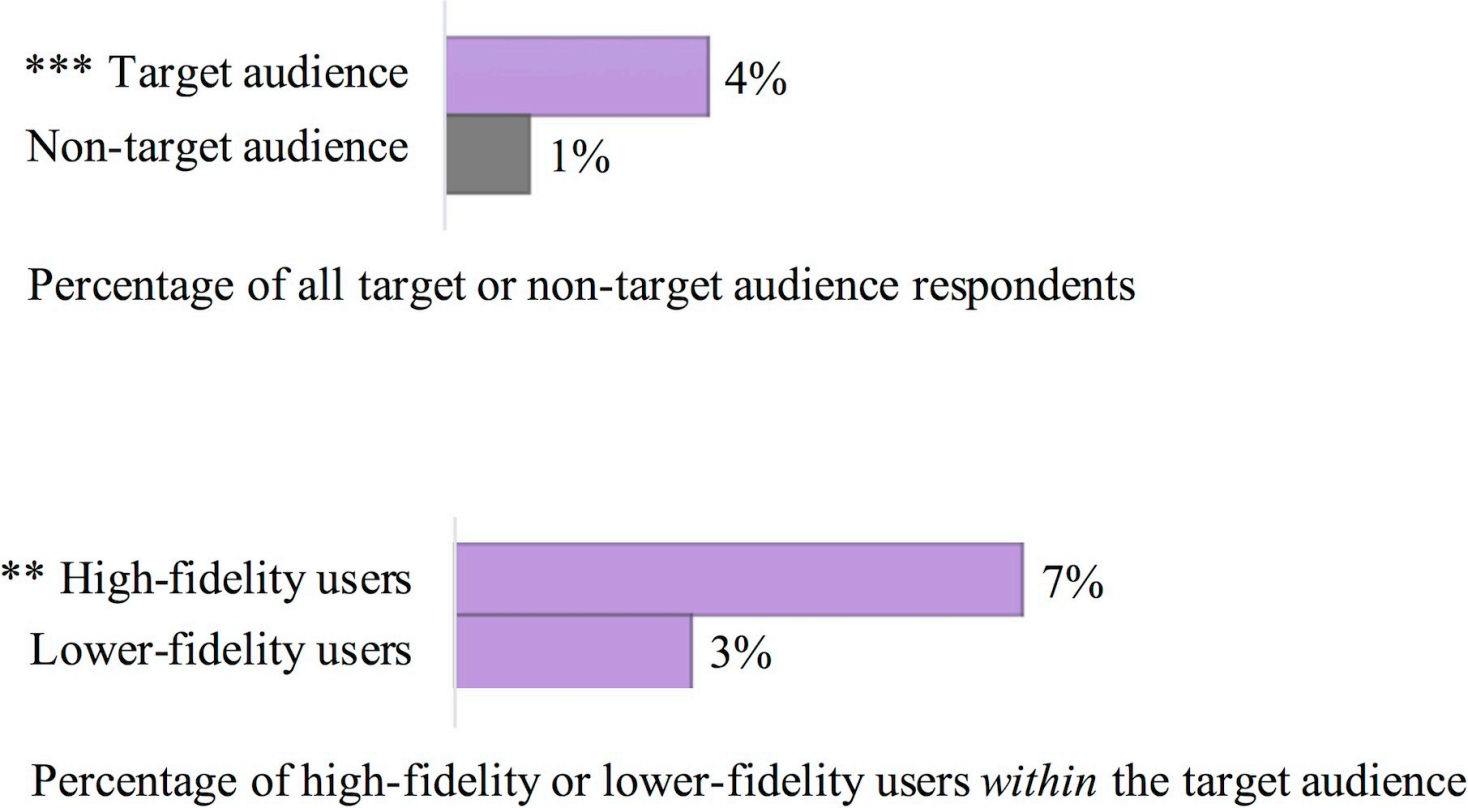
Stated decrease in saiga horn usage following the intervention for those with accurate intervention recall. Percentages of the top graph are out of the target or non-target audience respondents (438 and 1,678 people respectively). Percentages of the lower graph are out of high-fidelity and lower-fidelity users within the target audience (99 and 339 people respectively). Asterisks indicate the target audience and high-fidelity users within this audience were significantly more likely to state a behaviour change (** for p-value < 0.01, and *** for p-value < 0.001).

Regarding the intervention-specific questions in the post-intervention survey: among respondents with accurate intervention recall, 38 people (17 target audience respondents), stated that they had fully stopped or heavily decreased their saiga horn use (Fig 4). This value included both high-fidelity (14 people) and presumably lower-fidelity (24 people) saiga horn users. These lower-fidelity users are respondents who did not explicitly state in earlier questions that saiga horn was a product they use ‘most often’, nor that they had recently shifted away from saiga horn. But in later questions, these respondents indicated they did use saiga horn prior to the intervention. No respondents with accurate intervention recall stated that they increased their saiga horn usage. One non-target audience respondent with inaccurate message recall stated they intended to increase usage.

**Fig 5:**
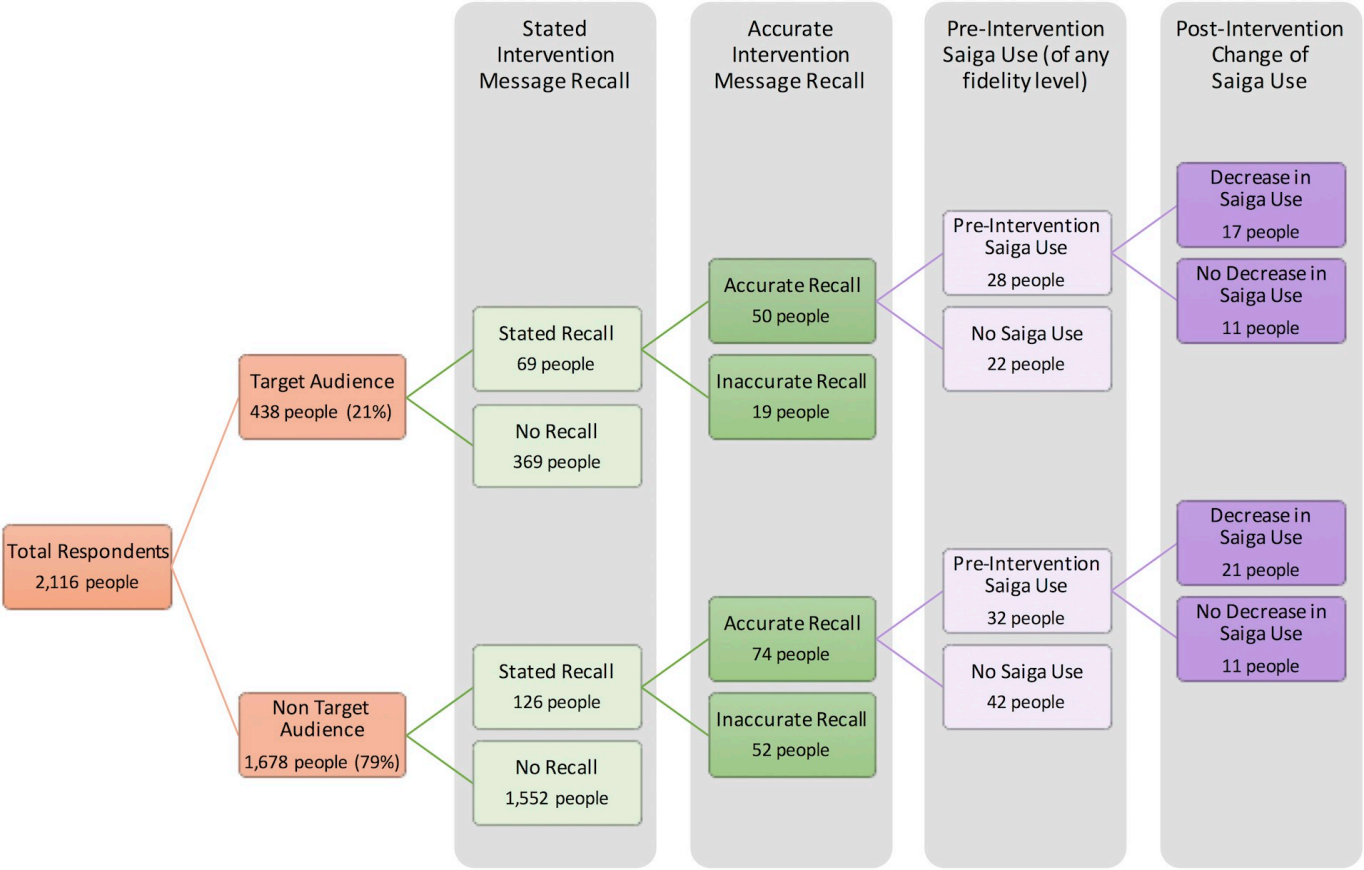
A funnel of consumer survey respondents from the total sample through till stated decrease in saiga horn usage following the intervention. Colours correspond to the Theory of Change stages outlined in Fig 1.

Target audience respondents were significantly more likely than non-target audience respondents to state they had decreased their saiga horn usage (4% versus 1%; ß = 1.19, z = 3.42, p = 6.3E^-4^; Fig 4). Within the target audience, high-fidelity users were significantly more likely than lower-fidelity users to state they had decreased their usage (7% versus 3%; ß = 1.11, z = 3.11, p = 0.0019; Fig 4). Fig 5 shows a more detailed view of intervention effect size by illustrating the funnelling from the total sample through those capable of being impacted by the intervention (i.e. saiga horn users) and those who were impacted by it (i.e. had accurate message recall and stated a behaviour change). Four percent (17 people) of target audience respondents stated a decrease in saiga horn usage, but as shown in Fig 5, this composes 61% of the 28 people who were previous saiga horn users in the target audience with accurate intervention message recall.

Among all respondents with accurate intervention recall who stated they had decreased their saiga horn use, the significantly most common reason given for changing behaviour was relevant to the intervention message: discussing the media attention specifically, the plight of the animal, relevant pro-environmental opinions, etc. (significance assessed in comparison to the next most common reason–a preference for alternative products– χ^2^(1, *N* = 38) = 19, *p* = 6.5E^-6^). The third most common reason given was saiga horn is ineffective. Among respondents with accurate intervention recall who stated they had not decreased their saiga horn use, the most common reason given was saiga horn’s efficacy (10 out of 22 people). Other stated reasons included that respondents felt they did not use saiga horn often, that they did not think their purchases alone made an impact, or that they did not care about the impact.

#### Shopkeeper survey

There was variation in perceived heatiness/fever treatment purchases between a shopkeeper’s target audience customers (mid-aged females) and their overall customer base (S5 File). They perceived target audience customers as purchasing animal products most (62%), and their overall customer base as purchasing herbal products most (55%). Saiga was overwhelmingly the most commonly purchased animal product, with 98% of both the target audience and overall customer base choosing saiga over other animal alternatives (goat, sheep, buffalo, ‘other’).

In the non-intervention specific questions, one shopkeeper stated they had seen a recent decrease in saiga horn purchases across customers, but they did not know why. Another shopkeeper stated that they had seen a recent decrease among the target audience and believed this was due to increasing prices of saiga horn products. There were no stated recent changes for other treatment types.

**Fig 6:**
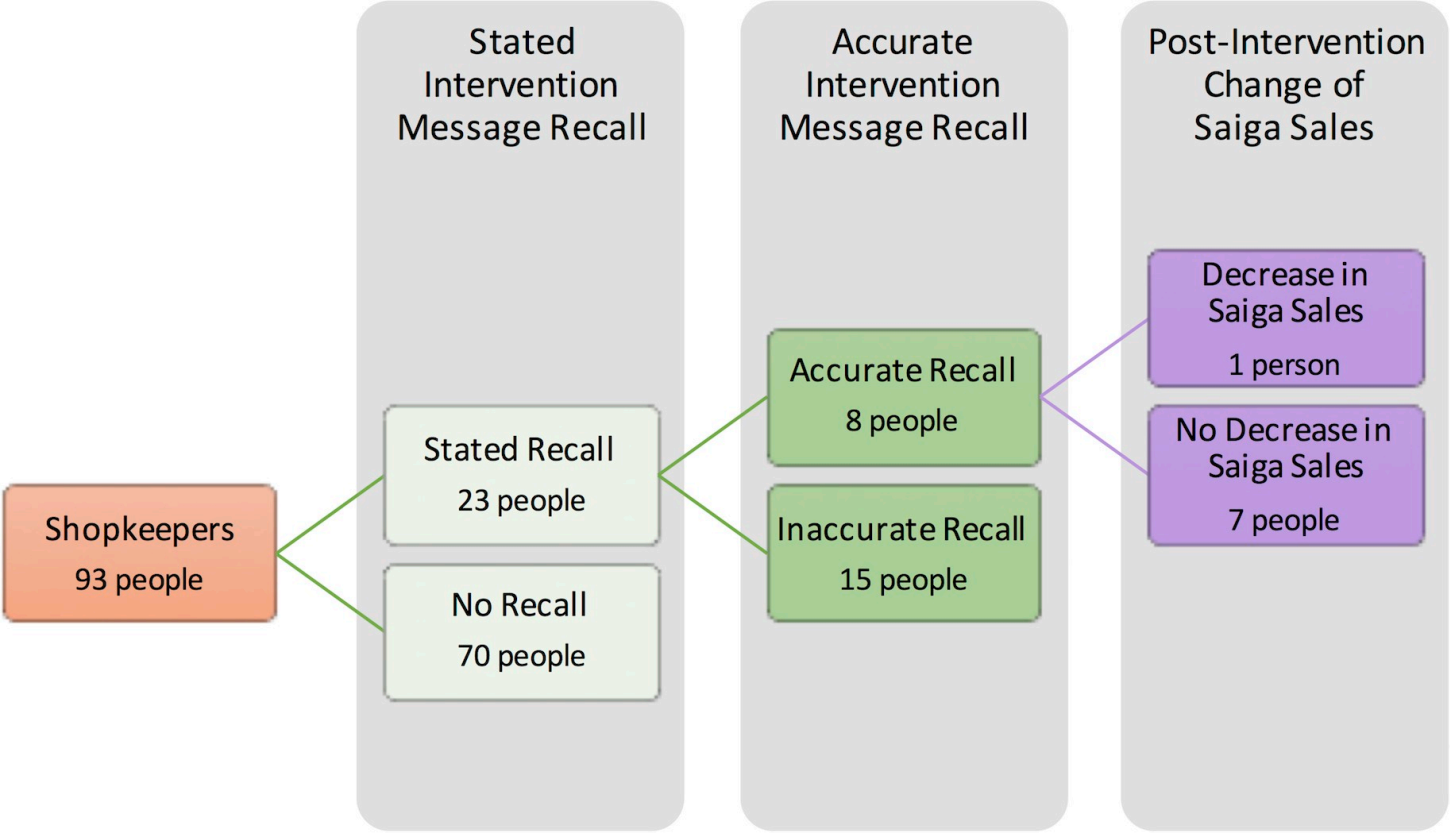
A funnel of shopkeeper respondents from the total respondent pool through till perceived decrease in saiga sales following the intervention. Colours correspond to the Theory of Change stages outlined in Fig 1.

In the intervention-specific questions: among those with accurate intervention recall, one shopkeeper perceived a decrease in saiga horn purchases across customers, including the target audience (Fig 6). They did not know the amount it decreased, but also said it was due to increasing saiga horn prices. Among those with an inaccurate message recall, four shopkeepers stated they had seen a decrease in saiga horn purchases, ranging from an unknown amount, to 25%, and more than 70%. They stated it was mainly due to saiga horn prices and changing customer preferences. Shopkeepers who stated there had been no sales change predominately discussed saiga horn’s efficacy, along with topics like tradition. No respondents mentioned an increase in saiga horn purchases.

Four shopkeepers with inaccurate intervention recall perceived there had been an increase in alternative products sales due to prices of saiga horn, convenience, the loss of tradition, and decreased imports of saiga horn. Further, three shopkeepers with inaccurate intervention recall said that they now recommend saiga horn less. Two said this was because of saiga horn prices and import regulations, while one shopkeeper cited decreasing saiga population numbers as their reason.

## Discussion

### Overview of impacts

The post-intervention target audience was significantly more likely than the non-target audience to report they had fully stopped (or heavily decreased) their saiga horn use following the intervention (4% versus 1%). We did not, however, find a significant decrease in frequency of high-fidelity saiga horn users from pre- to post-intervention (24.4% versus 22.6%). Among post-intervention respondents who did report a behaviour change, they gave intervention message-related reasons significantly most often. These findings were supported by the initial online analysis in which some individuals stated they intended to change their behaviour in response to the intervention message [24]. Additionally, across all consumer survey respondents there was a significant decrease from pre- to post-intervention in believing saigas are a common species in the wild, which was similarly supported by online responses indicating the message informed individuals of the saiga’s conservation status [24]. TCM shopkeepers, however, suggested that saiga horn sales trends were predominately related to price (rather than the intervention message).

### Unpacking the impact

It is worth exploring why our approach did not have a larger discernible impact on high-fidelity saiga horn users. One possibility is that for some saiga horn users, particularly high-fidelity users, their perception of saiga horn’s efficacy is a stronger driver of behaviour than external influences like social-level perceptions [25]. This consumer sub-group is thus not likely to be influenced by conservation or social messaging alone. Trying to shift their perception of saiga horn’s efficacy using biomedicine-based reasoning is also not likely to prove effective, given TCM users’ trust in their own experiences and the longevity of usage, as indicators of product efficacy [37, 38]. Consequently, this sub-group would probably be more affected by regulatory-type interventions that impact access to, or affordability of, saiga horn [39].

Another key possibility is that the study was under-scaled. The intervention design centred around the concept that individuals are more likely to adopt ideas they are exposed to repeatedly and in diverse ways [28]. Despite having notable online reach with high rates of engagement [24], it is *un*likely we reached a saturation point of repeatedly exposing the entire target audience within Singapore to the intervention message. Past research indicates there is variation in the number of repeated exposures that are necessary for different adverts to be effective [40]. As such, it is possible given a longer-running intervention, and thus increased target audience frequency of exposure, more of the nation’s target audience would have adopted the intervention message.

The timing of the post-intervention survey (two months after intervention completion) may have impacted results. Because saiga horn is only used when an individual is unwell, it is possible that even if a respondent’s perceptions of saiga horn had changed it is likely they may not yet have had a reason in that short time period to consider acting upon a newly formed perception, or to identify as a non-saiga horn user based on a changed behaviour choice.

#### Variability in saiga horn use

For our intervention we assumed that everyone who identified as a high-fidelity user had the same attachment to the product. However, conflicting statements about saiga horn use in our results (i.e. respondents selecting saiga horn as a product they use ‘most often’ but then stating they had actually switched to other products) indicates that not everyone who was categorised as a high-fidelity user may still use saiga horn all that often, or be attached to saiga horn products to the same extent. For example, we know that high-fidelity users are also likely to use traditional herbal products (S4 File), but the frequency with which they employ either treatment type is not known. Related, our treatment preference questions were designed to identify high-level saiga horn users, however, this choice meant we were unable to gauge temporal trends among more ‘peripheral’ users, even though our findings show that this lower-fidelity group did include people who stated that they had changed behaviour. All of which suggests future work should better segment consumer-groups by levels of usage, and gather psychographic information that would better unpack user fidelity to the product [41, 42].

### Methodological considerations

#### Response bias

There are a number of challenges around using recall to measure impact [18]. For instance, consumers may have known about the intervention message, or been affected by it, but may not have realised what we were referring to during the survey. Additionally, the finding that respondents with inaccurate message recall mostly cited family and friends as the message source, rather than social media and news, speaks to the diminished accuracy of word-of-mouth information [43]. Lastly, when recounting behaviour, it is expected that some individuals will inaccurately recall their own behaviour, or possibly downplay behaviour they think is socially undesirable [18]. In our study context saiga horn is non-sensitive to consumers, and we additionally strove to mitigate possible social pressure through question phrasing that would not prompt respondents into thinking that we, as researchers, wanted a particular answer regarding their behaviour (e.g. from value priming or social priming [44, 45]). There were a number of respondents with accurate intervention recall who still stated they were continuing to use saiga horn, and no consumer respondents seemed visibly uncomfortable or made comments to suggest they believed we were affiliated with, or promoting, the discussed media attention. Such observations suggest that respondents were not being prompted towards particular answers.

For our third-party reported dataset, we would have preferred quantitative sales data, however, this data was inaccessible so we conducted TCM shopkeeper surveys. These surveys proved challenging as many shopkeepers were busy, unwilling to answer questions, or keen to discuss off-topic narratives. For example, immediately after the researcher stated we were interested in recent media attention, many shopkeepers instead discussed old company textbooks, ‘years of experience’, etc. We also realised from the results that it might be difficult for shopkeepers to recognise or differentiate short-term changes from broader trends, especially since they did not have sales data to reference.

Additionally, Singaporeans do not always divulge their full medical choices to health professionals like TCM practitioners or biomedical doctors, for fear of disapproval [46, 47]. So even though our customers may take advice from TCM shopkeepers, they may not be willing to disclose their *own* reasons for selecting, or changing, purchase preferences. For example, consumers switching away from saiga horn may have cited more neutral reasons, like price, instead of value or norm-related reasons relating to wild animals, for fear it would be sensitive to shopkeepers with wild-derived products.

#### Possible confounders

As far as we are aware, there were no other saiga horn related interventions or major media attention running in Singapore between the baseline and evaluation periods that would confound attribution of our intervention’s impact. There was one news story about saiga horn seizures in China near to the evaluation time, but this was only mentioned by a single non-target audience respondent. There was also a letter sent out by the government to TCM shops during our 2017 surveys which reminded shopkeepers that saiga horn products require proper permits, but many shopkeepers had no recollection of this particular letter. There was mention among shopkeepers, though, of increased regulation and decreased availability as a general trend, which may be linked to government or industry communications like that letter, which we are not privy to, but which could impact shopkeepers’ sensitivity around discussing saiga horn, or their recommendation of the product to consumers.

### The bigger picture

The use of online social networking sites for public health goals is increasing, but many of these efforts have not been thoroughly evaluated [48]. Similarly, there have been numerous online efforts to increase sustainable consumption [49, 50]; however, few efforts have robustly assessed how their online work translates to offline behaviour change [51]. Furthermore, no other efforts directly targeting wildlife product consumers, to our knowledge, have utilised our approach of strategically spreading online news, nor evaluated this approach’s impact on offline behaviour.

Given the pitfalls that can occur in evaluating large-scale projects [52], we strove to use an evaluation design that was both robust and feasible. Since our goal was for country-level change and our baseline research incorporated demographic information, our target and non-target audiences were based on an entire demographic, rather than on who was exposed to the intervention, or who was definitively a saiga horn user. We also selected a cross-sectional design rather than a longitudinal one, as a longitudinal design at the country-level was cost-prohibitive [14].

If we relied solely on online performance metrics indicating intervention reach and immediate engagement (which are often the measures of success in wildlife trade interventions [2]) we would have over-estimated our intervention’s impact. Through our multi-pronged evaluation, we could more accurately discern how, and how not, the intervention was effective. Despite high public exposure and promising online engagement, we yielded only a small number of people who had decreased their saiga horn usage and who *explicitly* indicated this was due to the intervention. However, even in fields like public health, where interventions tend to be better funded, meta-analyses highlight that single interventions often have relatively small effect sizes [53, 54]. In addition, it is expected that behaviour change interventions will have heterogenous impacts on a target audience. Some implementers even argue that focusing on average impact per person, rather than total proportion of people impacted, is a more insightful gauge of an intervention’s success [15]. We thus need to be realistic in our expectations of unsustainable consumer intervention impacts and more nuanced in our understanding of them. In sum, our evaluation found the online intervention approach did impact perceptions and behaviour of consumers, and suggests that employment of this approach could benefit global sustainable consumption efforts. Though overall effect size, particularly on users of varying product fidelity, warrants improvement in future efforts.

Behaviour change interventions are iterative processes [9], and no study system is stagnant, nor identical to any other system. Consequently, regardless of an intervention’s impact, we should carefully and transparently document what was and was not effective, and what led to this outcome, so that future interventions can learn from these efforts. A recent review of behaviour change interventions on wildlife product consumers found that only 25% reported outcomes regarding behaviour or awareness changes resulting from an intervention [2]. Among this 25%, there was a dearth in transparency regarding evaluation methodology and robustness. Evaluation, though, affords the opportunity to use resources effectively by learning from past work, thereby provoking ever-adapting, larger, and more timely impacts.

## Supporting information

S1 File Additional survey methodology details. (PDF)

S2 File Full surveys. (XLSX)

S3 File Raw data. (XLSX)

S4 File Supplementary consumer results. (PDF)

S5 File Supplementary shopkeeper results. (PDF)
