# Supplementary material for: Evaluating a large-scale online behaviour change intervention aimed at wildlife product consumers in Singapore
Source: PLoS One. 2021 Mar 24;16(3):e0248144. doi: 10.1371/journal.pone.0248144 (PMC7990170; doi:10.1371/journal.pone.0248144)
Supplement: S1 File — (PDF) [file pone.0248144.s001.pdf]

S1 File: Additional Survey Methodology Details

Table of Contents

CONSUMER SURVEYS 2

Survey Approach *2*
Data Analyses *2*

SHOPKEEPERS SURVEYS 4

Survey Approach *4*
Data Analyses *5*

REFERENCES 5

Consumer Surveys

Survey Approach

Post-intervention (2019) consumer survey methodology heavily followed the same protocols detailed in the pre-intervention (2017) consumer surveys [1], so that the pre- and post-intervention surveys would be comparable.

Surveys were conducted in five Planning Areas (i.e. neighbourhood districts) in Singapore with the largest population of Chinese Singaporeans [2]. Chinese Singaporeans were targeted for this intervention because they are the largest consumers of complementary and alternative medicines (including TCM) in Singapore [3]. Within each Planning Area, we surveyed at four locations that would span patrons of varying socio-economic levels (Table 1). Survey collection was also stratified across day of week and time of day (weekday/weekend and morning-afternoon/afternoon-evening shifts).

Table 1. Consumer survey Planning Area and survey location.

Planning Area	Locations			
**Bedok**	Siglap Centre	Bedok Mall	Bedok South Food Centre	The Market Place @58
**Jurong West**	Jurong Point	Gek Poh Shopping Centre	Boon Lay Shopping Centre	Taman Jurong Shopping Centre
**Hougang**	Heartland Mall	Hougang Mall	Hougang Green Shopping Mall	Hougang 105 Hainanese Village Centre
**Tampines**	Tampines Mall	Eastpoint Mall	Hawker Centre 201	Prime Supermarket (Tampines Street 81)
**Sengkang**	Rivervale Mall	Compass One	The Seletar Mall	Kopitiam @ Block 275 D Compassvale Link

Surveyors wore red Nanyang Technological University (NTU) logo shirts and stated to potential respondents that the survey was asking heatiness and fever treatment preferences. There was no mention of saiga, TCM, wildlife, or the intervention. Surveyors also carried with them a letter of support detailing what the survey was about and indicating that it had ethics approval from both NTU and the University of Oxford. For respondents who elected to participate, the surveyor obtained informed consent as detailed by the ethics committees, prior to beginning the survey.

Surveyors asked every third person who passed them (while they were not with another respondent) to take the survey. If a group approached: surveyors asked the person on the right (for a group of two) or the third person to the right (for groups larger than two). The surveyor held the tablet the entire time, but stood next to the respondent so that the screen was clearly visible to them. The surveyor walked through each question orally in either English or Chinese, depending on the respondent's preference. The survey was also written in either English or Chinese. See Doughty et al. (2019) for more details.

Data Analyses

Statistical analyses followed the same approach as Doughty et al. (2019) where appropriate. For pre- and post-intervention comparisons we used the MatchIt package to match the 2017 and 2019 datasets, in order to account for possible sampling variances between years [4]. Specifically, we performed an 'optimal' match, with a 'mahalanobis' distance measure, and a 1:1 ratio. We chose optimal matching over the common 'nearest neighbour' matching, because optimal matching aims for the smallest average absolute distance across all the matched pairs, and has been found to be better than 'nearest neighbour' matching at minimising the distance within each pair [5]. Variables

matched included Chinese dialect, education, generation Singaporean, religion, and Target Audience (for the total sample comparisons). Comparisons of regressions (see below) between matched datasets and unmatched datasets did not yield any difference in variable significance, confirming that the characteristics of our post-intervention survey were quite similar to those of our pre-intervention survey.

We used General Linear Model regressions (GLMs) with sum contrasts applied [6], to assess differences between: pre- and post-intervention datasets, post-intervention target audience and other respondents, and post-intervention high-fidelity users and other respondents within the target audience. See Table 1 of the main Manuscript for GLM questions and variables used. We chose GLMs because they account for differences between the datasets, as well as variance caused by other demographic factors when comparing across groups. And we applied sum-contrasts to the GLMs because this allows the researcher to compare the mean of one variable level to the mean of all means across the levels within a variable [6]. In contrast, a default GLM output compares each variable's level to only one other 'reference' level within the variable.

All variables used in GLMs throughout this study can be seen in Table 2. Prior to running any GLMs, variable levels with less than 10 respondents were combined. The variable of 'Income' was omitted for the pre-post intervention comparisons as there was a 21% increase in number of respondents willing to disclose their income post-intervention, and we felt this discrepancy may inaccurately skew outputs. For our post-intervention specific comparisons, variable levels with less than 10 respondents for the target audience were omitted so they did not disproportionately skew results: 'Education Unknown', 'Religion Unknown', 'Religion Other', 'Dialect Unknown'.

Lastly, we used 2-sample z-tests for equality of proportions with a continuity correction to assess intervention message exposure sources, and reasons for behaviour change. This test was chosen because the respondent's answers on these questions were not mutually exclusive.

Table 2. Dependent and independent variables used in regression analyses throughout this study.

**Variable**	**Type** (as treated in the GLM)	**Levels**	**Description**
**Year**	Binary	2017<br>2019	Respondent was surveyed either pre- or post-intervention (in 2017 or 2019 respectively)
**Target Audience**	Binary	Yes<br>No	Respondent is, or is not, a female aged 35-59 years old
**High-fidelity saiga user**	Binary	Yes<br>No	Respondent did, or did not, state that saiga horn is a product they use 'most often' when treating fever/heatiness
**Chinese Dialect**	Categorical	Hokkiens<br>Teochews<br>Cantonese<br>Hakkas<br>Hainanese<br>Other<br>Prefer not to say	The Chinese dialect of the respondent's family
**Education**	Categorical	Pre-primary or primary school<br>Secondary school<br>ITE<br>Polytechnic<br>Junior college<br>Accredited diploma<br>University<br>Masters or PhD<br>Prefer not to say	Respondent's highest level of education

**Generation Singaporean**	Categorical	First<br>Second<br>Third<br>More than third<br>Prefer not to say	Generation Singaporean is the number of generations a respondent's family has lived in Singapore
**Income**	Categorical	Income 1 - Less than $5000<br>Income 2 - $5001 to $15,000<br>Income 3 - $15,001 to $25,000<br>Income 4 - $25,001 to $35,000<br>Income 5 - $35,001 and higher<br>Prefer not to say	Respondent's household average monthly income
**Religion**	Categorical	Buddhist<br>Muslim<br>Taoist<br>Catholic<br>Hindu<br>Christian (other)<br>Other<br>I do not identify with a religion<br>Prefer not to say	Respondent's religion

Shopkeepers Surveys

Survey Approach

Shopkeeper surveys were conducted in and around the same Planning Areas where the consumer surveys were conducted to allow for a rough comparability between the two (Table 3). There were five common shop types that we surveyed, as these sold saiga horn-like products (Table 3).

Table 3. Planning Areas and shop types visited in the shopkeeper surveys.

Planning Areas	Shop Types
Bedok +	TCM chain store
Geylang	Family-owned TCM store
Hougang +	TCM practitioner (some practitioners also sell TCM products)
Jurong East	Provision store (i.e. a store that sells a mix of food and home products)
Jurong West +	
Sengkang +	
Serangoon	
Tampines +	

+ Planning Area also used in the consumer surveys.

To begin the survey process, we identified possible TCM shops using a Search.insing.com business directory search, as well as Google Maps. We first visited shops in the five Planning Areas used in the consumer surveys, and as shops declined our surveys, we expanded this search to the surrounding Planning Areas.

The surveyor wore a red NTU logo shirt, and stated to the individual working behind the counter that they were conducting a study about which traditional fever and heatiness products customers purchase. There was no mention of saiga, TCM, wildlife, or the intervention. The surveyor also carried a letter of support detailing what the survey was about and indicating that it had ethics approval from both NTU and the University of Oxford. For shopkeepers who elected to participate, the surveyor obtained informed consent as detailed by the ethics committees, prior to beginning the survey.

Question phrasing was particularly important for the shopkeeper survey, and we carefully piloted and revised this survey in order to make shopkeepers feel as comfortable as possible. To ensure this

comfort, and not prompt the shopkeeper into thinking we were for or against the intervention, the respondent gave the following statement prior to asking about the intervention?

> "There are many factors that can influence or change a customer's choice of health products, such as their family's recommendations, media they see, price, and convenience. To understand the role of media better, for the next set of questions I am asking about the media reports from the past four months around ling yang, and your thoughts on how it may have affected your customer's choices."

Data Analyses

Due to the sample size of our shopkeeper survey, and the emphases on more qualitative answers, we chose to only do descriptive analyses. This was especially useful for the intervention-specific questions (e.g. assessing behaviour change following accurate intervention recall) when the sample size was below 10 respondents.

References

1. Doughty H, Veríssimo D, Tan RCQ, Lee JSH, Carrasco LR, Oliver K, et al. Saiga horn user characteristics, motivations, and purchasing behaviour in Singapore. PLOS ONE. 2019;14(9):e0222038.
2. URA (Urban Redevelopment Authority). Master Plan 2014. 2014 April 2019.
3. Lim MK, Sadarangani P, Chan HL, Heng JY Complementary and alternative medicine use in multiracial Singapore. Complementary Therapies in Medicine. 2005;13(1):16-24.
4. Ho D, Imai K, King G, Stuart E, Whitworth A Package MatchIt: Nonparametric Preprocessing for Parametric Causal Inference - Version 3.0.2. CRAN; 2018 January 09, 2018.
5. Ho DE, Imai K, King G, Stuart EA MatchIt: Nonparametric Preprocessing for Parametric Causal Inference. 2011 June 28, 2011.
6. Fox J An R and S-Plus companion to applied regression. Thousand Oaks, USA.; London, UK: Sage Publications; 2002.
