# Supplementary material for: Evaluating a large-scale online behaviour change intervention aimed at wildlife product consumers in Singapore
Source: PLoS One. 2021 Mar 24;16(3):e0248144. doi: 10.1371/journal.pone.0248144 (PMC7990170; doi:10.1371/journal.pone.0248144)
Supplement: S4 File — (PDF) [file pone.0248144.s004.pdf]

S4 File: Supplementary Consumer Survey Results

Table of Contents

CONSUMER DEMOGRAPHICS AND SAIGA USE **2**
Pre- and Post-Intervention Sample Demographics 2
Pre- and Post-Intervention High-Fidelity Frequency 3
PERCEPTIONS OF THE SAIGA'S CONSERVATION STATUS **5**
CONSUMER TREATMENT PREFERENCES **7**
Reasons, Recommenders, and Locations 7
Additional Treatment Types 9
INTERVENTION-SPECIFIC ANALYSES **9**
Accurate Intervention Recall 9
Sources of Exposure 12
Behaviour Change 13
Reasons for Behaviour Change 16
REFERENCES **17**

Consumer Demographics and Saiga Use

Pre- and Post-Intervention Sample Demographics

The post-intervention (2019) sample yielded a gender balance of 55% female, 45% male, and an age balance of 31% aged 18-34 years old, 35% aged 35-59 years old, and 34% aged 60+ years old. There were differences from 2017 to 2019 in the amount of demographic information respondents chose to share (Table 1). In 2019 about 5% more people chose to disclose their religion, dialect, education, and generation; and a stark 21% more people shared their income.

Table 1: Comparing Pre- and Post-Intervention (2017 and 2019) sample demographic proportions for the respondents used in analyses on saiga consumers.

**Age**							
	Young	*Mid-age*	*Old*				
2017	34%	34%	31%				
2019	31%	35%	34%				
**Sex**							
	Female	Male					
2017	51%	49%					
2019	55%	45%					
**Dialect**							
	Teochews	*Cantonese*	*Hainanese*	*Hakkas*	*Hokkiens*	*Other dialect*	*Did not say*
2017	21%	15%	5%	8%	41%	4%	6%
2019	20%	17%	6%	8%	42%	6%	1%
**Education**							
	Primary	*Secondary/ ITE*	*Pre-uni/ Post-sec*	*Uni/Grad*	*Did not say*		
2017	15%	27%	25%	25%	7%		
2019	13%	26%	28%	31%	2%		
**Generation Singaporean**							
	First	*Second*	*Third*	*More than third*	*Did not say*		
2017	22%	40%	23%	6%	9%		
2019	17%	30%	38%	11%	4%		
**Religion**							
	Buddhist	*Taoist*	*Catholic*	*Christian*	*No religion*	*Other religion*	*Did not say*
2017	42%	5%	6%	15%	25%	1%	5%
2019	40%	8%	6%	19%	25%	1%	1%
**Income**							
	Income one	*Income two*	*Income three*	*Income four*	*Income five*	*Did not say*	
2017	31%	20%	5%	2%	3%	39%	
2019	40%	26%	7%	3%	6%	18%	

Pre- and Post-Intervention High-Fidelity Frequency

Sample year (2017 versus 2019) was not significantly associated with high-fidelity saiga horn use across the total sample or the target audience (Tables 2 and 3). In other words, high-fidelity use was *not* more likely to occur in a given year.

Table 2. GLM Output for profiles of respondents indicating high-fidelity saiga use pre- and post-intervention, across the total sample. Sum contrasts have been applied, in which "each coefficient compares the corresponding level of the factor to the average of the other levels" [1].

Did high-fidelity saiga horn use, across the total samples, change from 2017 to 2019?

No - Not Significant

High-fidelity saiga use = year - target-audience + Chinese dialect + education + generation Singaporean + religion

((the variable 'income' was omitted because 20% more people disclosed their income in 2019 than in 2017 and we felt this discrepancy may disproportionately impact outputs))

Coefficients:	Estimate	Standard Error	Z-Value	Pr(>|z|)	
(Intercept)	-1.614	0.104	-15.523	0.000	***
Year 2019	-0.088	0.093	-0.938	0.348	
Target Audience	0.325	0.135	2.405	0.016	*
Year - Target Audience Interaction	0.042	0.190	0.221	0.825	
Dialect Cantonese	0.011	0.109	0.096	0.923	
Dialect Hainanese	-0.060	0.173	-0.347	0.729	
Dialect Hakkas	0.421	0.130	3.236	0.001	**
Dialect Hokkiens	0.237	0.084	2.806	0.005	**
Dialect Teochews	0.102	0.101	1.013	0.311	
Dialect Other	-0.301	0.198	-1.523	0.128	
Dialect Unknown	-0.409	0.265	-1.547	0.122	
Education Primary School (and Under)	0.282	0.093	3.014	0.003	**
Education Secondary School / ITE	-0.101	0.080	-1.258	0.208	
Education Pre-University / Post-Secondary School	-0.259	0.084	-3.094	0.002	**
Education University / Graduate School	-0.111	0.083	-1.336	0.182	
Education Unknown	0.189	0.167	1.132	0.258	
Generation First	-0.147	0.090	-1.642	0.101	
Generation Second	-0.099	0.075	-1.323	0.186	
Generation Third	-0.027	0.079	-0.339	0.735	
Generation More than Third	0.255	0.117	2.184	0.029	*
Generation Unknown	0.018	0.148	0.119	0.905	
Religion Buddhist	0.354	0.101	3.516	0.000	***
Religion Catholic	-0.311	0.181	-1.720	0.085	.
Religion Christian	-0.024	0.123	-0.197	0.843	
Religion Taoist	0.347	0.149	2.329	0.020	*
Religion None	-0.253	0.116	-2.181	0.029	*
Religion Other	-0.296	0.382	-0.775	0.438	
Religion Unknown	0.184	0.268	0.687	0.492	

```
---
Signif. codes:  0 ‘***’ 0.001 ‘**’ 0.01 ‘*’ 0.05 ‘.’ 0.1 ‘ ’ 1
Null deviance: 4093.4  on 4207  degrees of freedom
Residual deviance: 3975.6  on 4184  degrees of freedom
AIC: 4023.6
Number of Fisher Scoring iterations: 4
```

Table 3. GLM Output for profiles of respondents indicating high-fidelity saiga use pre- and post-intervention, within the target audience. Sum contrasts have been applied, in which “each coefficient compares the corresponding level of the factor to the average of the other levels” [1].

Did high-fidelity saiga horn use, in the target audience, change from 2017 to 2019?

No - Not Significant

High-fidelity saiga use = year + Chinese dialect + education + generation Singaporean + religion

((the variable ‘income’ was omitted because 20% more people disclosed their income in 2019 than in 2017 and we felt this discrepancy may disproportionately impact outputs))

Coefficients:	Estimate	Standard Error	Z-Value	Pr(>|z|)	
(Intercept)	-1.272	0.237	-5.371	0.000	***
Year 2019	-0.106	0.171	-0.617	0.537	
Dialect Cantonese	0.102	0.219	0.464	0.643	
Dialect Hainanese	0.006	0.367	0.016	0.987	
Dialect Hakkas	-0.396	0.324	-1.224	0.221	
Dialect Hokkiens	0.368	0.176	2.097	0.036	*
Dialect Teochews	0.056	0.210	0.269	0.788	
Dialect Other	0.263	0.374	0.703	0.482	
Dialect Unknown	-0.399	0.560	-0.713	0.476	
Education Primary School (and Under)	0.433	0.234	1.847	0.065	.
Education Secondary School / ITE	-0.035	0.172	-0.207	0.836	
Education Pre-University / Post-Secondary School	-0.104	0.180	-0.580	0.562	
Education University / Graduate School	-0.239	0.183	-1.308	0.191	
Education Unknown	-0.054	0.425	-0.127	0.899	
Generation First	-0.188	0.198	-0.948	0.343	
Generation Second	-0.127	0.155	-0.818	0.413	
Generation Third	-0.194	0.158	-1.231	0.218	
Generation More than Third	0.134	0.243	0.549	0.583	
Generation Unknown	0.375	0.317	1.182	0.237	
Religion Buddhist	0.213	0.243	0.877	0.381	
Religion Catholic	0.248	0.347	0.713	0.476	
Religion Christian	0.156	0.269	0.580	0.562	
Religion Taoist	0.043	0.367	0.116	0.908	
Religion None	0.182	0.262	0.693	0.488	
Religion Other	0.027	0.983	0.028	0.978	
Religion Unknown	-0.869	0.695	-1.251	0.211	

Signif. codes: 0 ‘***’ 0.001 ‘**’ 0.01 ‘*’ 0.05 ‘.’ 0.1 ‘ ’ 1
Null deviance: 955.60 on 875 degrees of freedom
Residual deviance: 932.96 on 854 degrees of freedom
AIC: 976.96
Number of Fisher Scoring iterations: 4

Perceptions of the Saiga’s Conservation Status

Respondents were asked which animals (out of a list of animals used often in TCM) were common in the wild. Responses pre- and post-intervention are shown in Table 4. Sample year (2017 versus 2019) was significantly associated with misidentifying saiga antelope as common, for both the total sample and the target audience (Tables 5 and 6). In other words, misperceptions that saiga antelopes are a common species in the wild decreased significantly from 2017 to 2019.

Table 4. Wild Animals perceived as common in the wild. Frequencies and percentages out of total respondents for each sample (2,294 pre-intervention; 2,116 post-intervention) are shown.

	2017		2019	
Sea cucumber (hai shen)	844	(37%)	879	(42%)
Goat (shan yang)	871	(38%)	722	(34%)
Turtle (hai gui)	649	(28%)	606	(29%)
Saiga antelope (ling yang)	641	(28%)	452	(21%)
Sea horse (hai ma)	621	(27%)	556	(26%)
Rhino (xi niu)	343	(15%)	313	(15%)
None -- N/A in 2017	(0	(0%)	349	(16%)
I don't know	632	(28%)	449	(21%)

Table 5: GLM output for profiles of respondents who misidentified saiga antelopes as being common in the wild, pre- and post-intervention. Sum contrasts have been applied, in which “each coefficient compares the corresponding level of the factor to the average of the other levels” [1].

Did misconceptions of saigas being common in the wild, across the total samples, change from 2017 to 2019?

Yes - Significant

Misidentifying saiga as common in the wild = year - target-audience + Chinese dialect + education + generation Singaporean + religion

((the variable ‘income’ was omitted because 20% more people disclosed their income in 2019 than in 2017 and we felt this discrepancy may disproportionately impact outputs))

Coefficients:	Estimate	Standard Error	Z-Value	Pr(>|z|)	
(Intercept)	-1.170	0.092	-12.669	0.000	***
Year 2019	-0.439	0.084	-5.224	0.000	***
Target Audience	0.188	0.122	1.545	0.123	
Year - Target Audience Interaction	0.117	0.175	0.669	0.504	
Dialect Cantonese	0.132	0.094	1.401	0.161	
Dialect Hainanese	-0.323	0.161	-2.008	0.045	*
Dialect Hakkas	0.288	0.120	2.407	0.016	*

Dialect Hokkiens	0.117	0.075	1.549	0.121	
Dialect Teochews	0.165	0.089	1.862	0.063	.
Dialect Other	0.168	0.149	1.126	0.260	
Dialect Unknown	-0.547	0.232	-2.355	0.019	*
Education Primary School (and Under)	-0.063	0.095	-0.667	0.505	
Education Secondary School / ITE	-0.023	0.075	-0.302	0.762	
Education Pre-University / Post-Secondary School	0.158	0.074	2.146	0.032	*
Education University / Graduate School	0.084	0.076	1.105	0.269	
Education Unknown	-0.156	0.164	-0.954	0.340	
Generation First	-0.213	0.084	-2.553	0.011	*
Generation Second	-0.004	0.068	-0.059	0.953	
Generation Third	0.057	0.071	0.794	0.427	
Generation More than Third	-0.150	0.115	-1.311	0.190	
Generation Unknown	0.311	0.132	2.360	0.018	*
Religion Buddhist	0.094	0.091	1.037	0.300	
Religion Catholic	0.009	0.145	0.065	0.949	
Religion Christian	0.079	0.107	0.740	0.460	
Religion Taoist	0.284	0.138	2.059	0.040	*
Religion None	0.066	0.098	0.673	0.501	
Religion Other	-0.212	0.324	-0.655	0.513	
Religion Unknown	-0.321	0.257	-1.247	0.212	

Signif. codes: 0 '***' 0.001 '**' 0.01 '*' 0.05 '.' 0.1 ' ' 1

Null deviance: 4710.5 on 4207 degrees of freedom

Residual deviance: 4633.2 on 4184 degrees of freedom

AIC: 4681.2

Number of Fisher Scoring iterations: 4

Table 6. GLM output for profiles of respondents who misidentified saiga antelopes as being common in the wild, pre- and post-intervention, within the target audience. Sum contrasts have been applied, in which "each coefficient compares the corresponding level of the factor to the average of the other levels" [1].

Did misconceptions of saigas being common in the wild, in the target audience, change from 2017 to 2019?

Yes - Significant

Misidentifying saiga as common in the wild = year + Chinese dialect + education + generation Singaporean + religion

((the variable 'income' was omitted because 20% more people disclosed their income in 2019 than in 2017 and we felt this discrepancy may disproportionately impact outputs))

Coefficients:	Estimate	Standard Error	Z-Value	Pr(>|z|)	
(Intercept)	-0.774	0.205	-3.785	0.000	***
Year 2019	-0.363	0.161	-2.250	0.024	*
Dialect Cantonese	0.168	0.197	0.851	0.395	
Dialect Hainanese	-0.299	0.362	-0.825	0.410	
Dialect Hakkas	0.266	0.256	1.036	0.300	

Dialect Hokkiens	0.070	0.164	0.425	0.671	
Dialect Teochews	0.074	0.190	0.392	0.695	
Dialect Other	0.343	0.341	1.005	0.315	
Dialect Unknown	-0.622	0.490	-1.269	0.204	
Education Primary School (and Under)	-0.184	0.242	-0.761	0.447	
Education Secondary School / ITE	-0.225	0.166	-1.357	0.175	
Education Pre-University / Post-Secondary School	0.213	0.165	1.292	0.197	
Education University / Graduate School	0.115	0.168	0.684	0.494	
Education Unknown	0.081	0.390	0.209	0.835	
Generation First	-0.400	0.198	-2.024	0.043	*
Generation Second	0.104	0.149	0.699	0.485	
Generation Third	0.053	0.151	0.355	0.723	
Generation More than Third	-0.136	0.250	-0.544	0.586	
Generation Unknown	0.379	0.315	1.202	0.229	
Religion Buddhist	0.000	0.211	0.001	0.999	
Religion Catholic	-0.257	0.316	-0.813	0.416	
Religion Christian	0.031	0.231	0.136	0.892	
Religion Taoist	0.353	0.315	1.119	0.263	
Religion None	-0.222	0.233	-0.952	0.341	
Religion Other	0.681	0.812	0.838	0.402	
Religion Unknown	-0.586	0.561	-1.044	0.296	

Signif. codes: 0 ‘***’ 0.001 ‘**’ 0.01 ‘*’ 0.05 ‘.’ 0.1 ‘ ’ 1

Null deviance: 1054.9 on 875 degrees of freedom

Residual deviance: 1028.9 on 854 degrees of freedom

AIC: 1080.5

Number of Fisher Scoring iterations: 4

Consumer Treatment Preferences

Reasons, Recommenders, and Locations

Post-intervention (in 2019) we saw a number of statistically significant trends among high-fidelity saiga horn consumers using saiga horn on themselves, which were mainly in keeping with our Pre-intervention (2017) results (Fig 1). Firstly, *it works* and *someone recommended it to me* remained the top two stated reasons for using saiga horn, and *family* remained the top recommender of saiga horn (though the second recommender shifted from *TCM shopkeeper* in 2017 to *friend* in 2019). Next, *TCM stores* remained the top location to buy saiga horn products (however the order flipped between *family-owned stores* and *chain stores*). Finally, high-fidelity saiga horn users were still less likely to also use biomedical products to treat heatiness and fever, and more likely to also use traditional herbal products. Statistical outputs shown below.

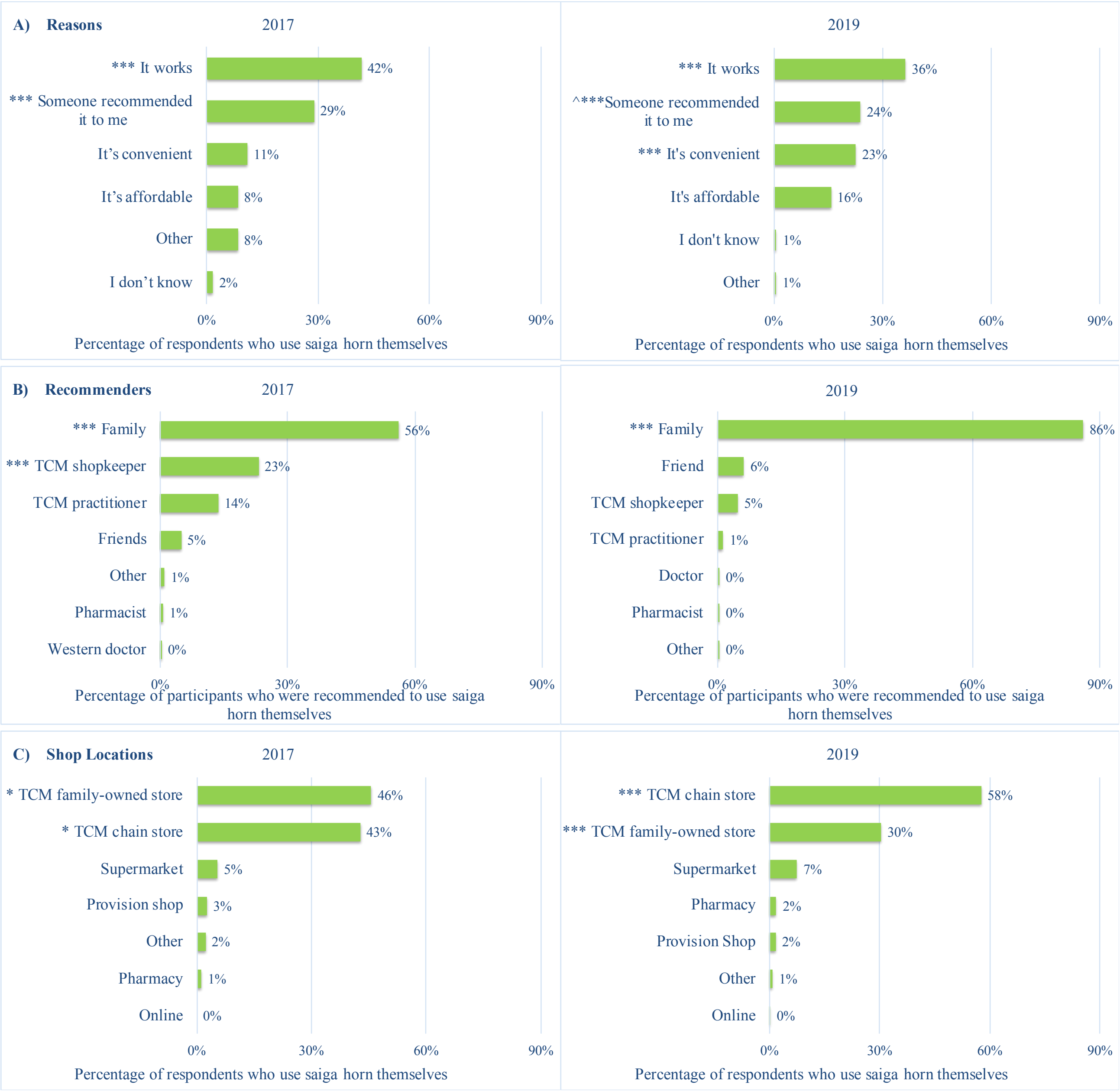

Figure 1. Trends for those using saiga horn to treat fever/heatiness in themselves Pre- and post-intervention (2017 and 2019). **A)** Reasons for using saiga horn stated by respondents. **B)** Recommenders for using saiga horn stated by respondents. **C)** Shop locations for purchasing saiga horn stated by respondents. Astride indicate: * is p < 0.05, ** is p < 0.01, *** p < 0.001. See Doughty et al. (2019) for 2017 statistics.

2-sample test for equality of proportions with continuity correction:

Reasons for Using Saiga

"It works" vs "Someone recommended it to me"

data: c(286, 188) out of c(343, 343)

X-squared = 64.232, df = 1, p-value = 5.529e-16

"Someone recommended it to me" vs "It's Convenient"

data: c(188, 178) out of c(343, 343)

X-squared = 0.47444, df = 1, p-value = 0.2455

"It's Convenient" vs "It's Affordable"
data: c(178, 124) out of c(343, 343)
X-squared = 16.616, df = 1, p-value = 2.288e-05

Recommenders of Saiga Horn
"Family" vs "Friend"
data: c(178, 13) out of c(188, 188)
X-squared = 286.2, df = 1, p-value < 2.2e-16

Locations for Purchasing Saiga Horn
"TCM Chain Store" vs "TCM Family Store"
data: c(277, 146) out of c(343, 343)
X-squared = 104.21, df = 1, p-value < 2.2e-16

"TCM Family Store" vs "Supermarket"
data: c(146, 36) out of c(343, 343)
X-squared = 88.854, df = 1, p-value < 2.2e-16

Additional Treatment Types

Different from our pre-intervention sample (2017), in our post-intervention sample (2019): among those using saiga horn on themselves, respondents were significantly more likely to also use traditional herbal medicine, and significantly less likely to also use western or other medicine (Table 7).

Table 7. Comparing the additional treatment types that those who saiga horn on themselves, Pre- and post-intervention (2017 and 2019).

	**2017**					**2019**			
	ß	Std. Error	Z-value	P-value ^		ß	Std. Error	Z-value	P-value
Intercept	0.08621	0.19248	0.448		Intercept	-1.298	0.248	-5.239	***
Western Med	-1.3304	0.14983	-8.879	***	Western Med	-0.906	0.187	-4.856	***
Herbal Trad Med	-0.1913	0.13378	-1.43		Herbal Trad Med	0.794	0.168	4.741	***
Other	-1.51281	0.16556	-9.138	***	Other	-0.904	0.202	-4.480	***

Intervention-Specific Analyses

Accurate Intervention Recall

Respondents mentioned a number of topics of when describing what they thought the "recent media attention about ling yang" was about (Table 8). The target audience, and high fidelity saiga horn users within this audience, were significantly more likely to describe content generally in-line with the intervention recall (and thus to be identified as having 'accurate intervention recall') (Tables 9 and 10).

Table 8. Stated recall, and the "media attention" content described by respondents. 'Saiga as an Animal' content refers to generally accurate descriptions of the intervention message. Frequencies and percentages out of the non-target and target audience (1678 and 438 people respectively) are shown.

	Non target audience		Target audience	
Stated recall	126	(8%)	69	16%
Saiga as an Animal (accurate)	74	(4%)	50	(11%)
Saiga horn not effective	16	(1%)	7	(2%)
Saiga horn effective	35	(2%)	12	(3%)

Saiga horn products are fake/diluted	4	(0%)	2	(0%)
Saiga horn Seizures	2	(0%)	2	(0%)
Generically negative towards saiga horn	2	(0%)	5	(1%)
I don't know	6	(0%)	0	(0%)

Table 9. GLM output for profiles of respondents who accurately recalled the intervention message. Sum contrasts have been applied, in which "each coefficient compares the corresponding level of the factor to the average of the other levels" [1].

Did the 2019 target audience accurately recall the intervention more than the non-target audience?					
Yes - Significant					
**Accurate intervention message recall = target-audience + Chinese dialect + education + generation Singaporean + religion + income** ((variable levels with less than 10 participants for the target audience were omitted so they did not disproportionately skew results: 'Education Unknown', 'Religion Unknown', 'Religion Other', 'Dialect Unknown'))					
Coefficients:	Estimate	Standard Error	Z-Value	Pr(>|z|)	
(Intercept)	-3.513	0.254	-13.854	< 2.00E-16	***
Target Audience	0.943	0.201	4.688	0.000	***
Dialect Cantonese	0.127	0.228	0.556	0.578	
Dialect Hainanese	-0.399	0.402	-0.992	0.321	
Dialect Hakkas	0.114	0.301	0.377	0.706	
Dialect Hokkiens	0.030	0.178	0.167	0.868	
Dialect Teochews	-0.159	0.231	-0.689	0.491	
Dialect Other	0.288	0.318	0.904	0.366	
Education Primary School (and Under)	-0.843	0.398	-2.118	0.034	*
Education Secondary School / ITE	0.078	0.206	0.379	0.705	
Education Pre-University / Post-Secondary School	0.027	0.214	0.124	0.901	
Education University / Graduate School	0.738	0.192	3.847	0.000	***
Generation First	-0.171	0.252	-0.677	0.499	
Generation Second	-0.264	0.211	-1.247	0.212	
Generation Third	0.213	0.176	1.209	0.226	
Generation More than Third	0.034	0.273	0.126	0.900	
Generation Unknown	0.187	0.390	0.479	0.632	
Religion Buddhist	0.279	0.183	1.524	0.127	
Religion Catholic	-0.125	0.341	-0.367	0.714	
Religion Christian	-0.356	0.239	-1.488	0.137	
Religion Taoist	-0.223	0.389	-0.575	0.565	
Religion None	0.425	0.195	2.179	0.029	*
Income One	-0.110	0.216	-0.508	0.611	
Income Two	0.247	0.190	1.296	0.195	
Income Three	0.327	0.272	1.205	0.228	
Income Four	-0.293	0.510	-0.575	0.565	

Income Five	0.236	0.292	0.806	0.420	
Income Unknown	-0.407	0.272	-1.497	0.134	

Signif. codes: 0 ‘***’ 0.001 ‘**’ 0.01 ‘*’ 0.05 ‘.’ 0.1 ‘ ’ 1

Null deviance: 908.49 on 2004 degrees of freedom

Residual deviance: 833.71 on 1982 degrees of freedom

AIC: 879.71

Number of Fisher Scoring iterations: 16

Table 10. GLM output for profiles of respondents who accurately recalled the intervention message, within the target audience. Sum contrasts have been applied, in which “each coefficient compares the corresponding level of the factor to the average of the other levels” [1].

Did 2019 high-fidelity users in the target audience accurately recall the intervention more than others in the target audience?

Yes - Significant

Accurate intervention message recall = high-fidelity saiga user + Chinese dialect + education + generation Singaporean + religion + income

((variable levels with less than 10 participants for the target audience were omitted so they did not disproportionately skew results: ‘Education Unknown’, ‘Religion Unknown’, ‘Religion Other’, ‘Dialect Unknown’))

Coefficients:	Estimate	Standard Error	Z-Value	Pr(>|z|)	
(Intercept)	-3.355	0.248	-13.504	< 2.00E-16	***
High-Fidelity Saiga User	0.456	0.226	2.023	0.043	*
Dialect Cantonese	0.104	0.226	0.459	0.646	
Dialect Hainanese	-0.354	0.400	-0.885	0.376	
Dialect Hakkas	0.078	0.301	0.258	0.797	
Dialect Hokkiens	0.021	0.176	0.122	0.903	
Dialect Teochews	-0.159	0.230	-0.692	0.489	
Dialect Other	0.310	0.317	0.978	0.328	
Education Primary School (and Under)	-0.934	0.398	-2.347	0.019	*
Education Secondary School / ITE	0.129	0.205	0.631	0.528	
Education Pre-University / Post-Secondary School	0.046	0.213	0.217	0.828	
Education University / Graduate School	0.758	0.192	3.954	0.000	***
Generation First	-0.108	0.251	-0.430	0.667	
Generation Second	-0.248	0.211	-1.176	0.240	
Generation Third	0.251	0.175	1.437	0.151	
Generation More than Third	-0.026	0.272	-0.096	0.924	
Generation Unknown	0.131	0.389	0.336	0.737	
Religion Buddhist	0.273	0.182	1.502	0.133	
Religion Catholic	-0.142	0.339	-0.418	0.676	
Religion Christian	-0.268	0.236	-1.136	0.256	
Religion Taoist	-0.257	0.386	-0.665	0.506	
Religion None	0.394	0.194	2.029	0.043	*

Income One	-0.141	0.215	-0.655	0.512
Income Two	0.232	0.190	1.226	0.220
Income Three	0.415	0.269	1.543	0.123
Income Four	-0.309	0.510	-0.605	0.545
Income Five	0.223	0.291	0.768	0.443
Income Unknown	-0.422	0.271	-1.556	0.120

Signif. codes: 0 ‘***’ 0.001 ‘**’ 0.01 ‘*’ 0.05 ‘.’ 0.1 ‘ ’ 1

Null deviance: 908.49 on 2004 degrees of freedom

Residual deviance: 850.50 on 1982 degrees of freedom

AIC: 896.5

Number of Fisher Scoring iterations: 6

Sources of Exposure

Respondents were asked where they heard about the “recent media attention on ling yang” (Fig 2). Those with accurate intervention recall were significantly more likely to cite the news and social media, while those with inaccurate intervention recall were significantly more likely to cite family and friends. Statistical outputs shown below.

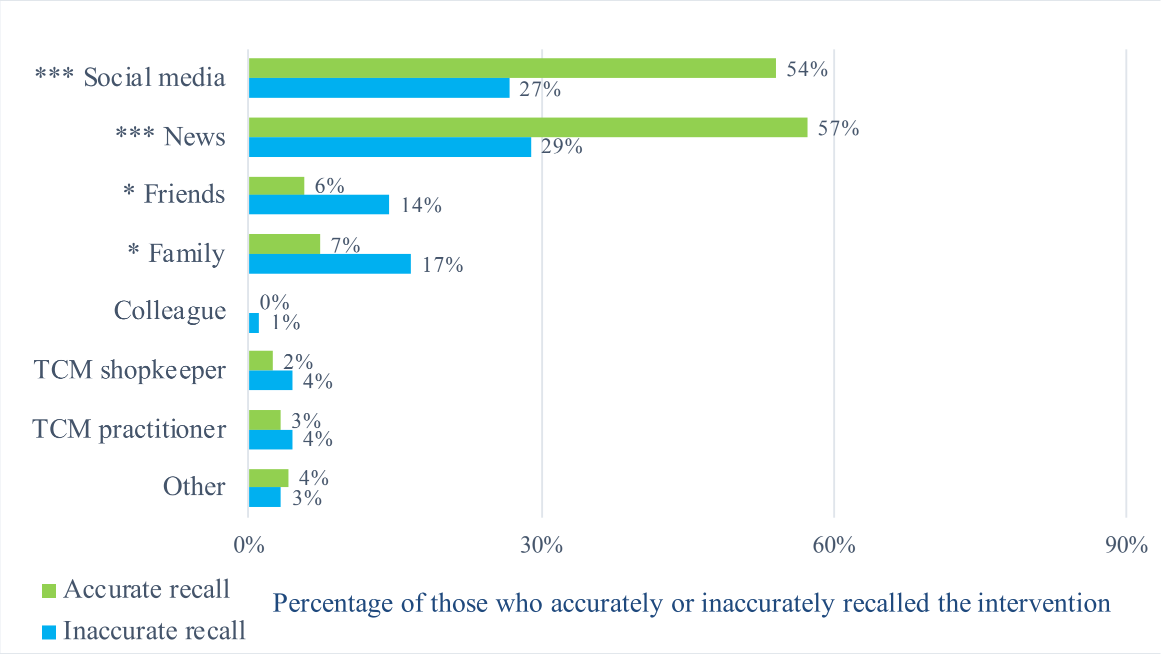

Figure 2. Sources of exposure to the intervention message. Percentages out of all respondents with accurate or inaccurate intervention message recall.

2-sample test for equality of proportions with continuity correction:

News

“Accurate recall” vs “Inaccurate recall”

data: c(71, 26) out of c(124, 90)

X-squared = 15.811, df = 1, p-value = 3.5e-05

Social Media

"Accurate recall" vs "Inaccurate recall"

data: c(67, 24) out of c(124, 90)

X-squared = 14.879, df = 1, p-value = 5.733e-05

Family

"Inaccurate recall" vs "Accurate recall"

data: c(15, 9) out of c(90, 124)

X-squared = 3.7395, df = 1, p-value = 0.02657

Friends

"Inaccurate recall" vs "Accurate recall"

data: c(13, 7) out of c(90, 124)

X-squared = 3.7839, df = 1, p-value = 0.02587

Behaviour Change

There were two ways that we assessed behaviour change following the intervention message. Firstly, we asked respondents if their fever/heatiness treatment preferences had changed in the last four months for any reason, with no mention of the intervention. Nine people who selected saiga as a product they purchase most often (and thus were categorised as high-fidelity saiga users), when next asked if their preferences changed for any reason in the last three months, said yes and either referenced herbal alternatives they are switching to or specifically referenced the news/Facebook posts (Table 11). Secondly, after asking respondents whether they recalled recent media attention around ling yang (saiga horn), we asked them if they had changed their usage of saiga horn. Among those with accurate intervention recall, target audience respondents were significantly more likely than non-target audience respondents to state they had decreased their saiga horn usage (Table 12). And within the target audience, high-fidelity saiga horn users were significantly more likely than presumably lower-fidelity users to state they had decreased their saiga horn usage (Table 13).

Table 11. Responses given by respondents who indicated high-fidelity saiga horn use, but then stated they had in fact changed their preference in the last four months.

Heatiness or Fever	Target Audience	Responses as they were recorded by the research surveyors
H		"Use more herbal product instead because it's more convenient"
H		"News"
H	Y	"Last used ling yang last year, will opt for herbal alternatives like barley if heaty now"
H		"There is other options like food and fruits"
H & F	Y	"Found ling yang so cooling that respondent has leg pain. Also read a Facebook post on how ling yang is harmed for their horns, feel pity towards them so haven't drink or buy ling yang this year. So now use more of herbal alternatives like barley water"
H		"If there is no need, I purchase water instead of ling yang if heaviness occurs."
F		"Became vegan in the last 2 months but haven't fallen sick during this period; would probably not use ling yang anymore if still maintaining diet the next time she gets fever"
F		"If ling yang doesn't work, respondent will take Panadol."
F	Y	"If these three methods does not work, the respondent will eat lots of fruits and drink plenty of water."

Table 12. GLM output for profiles of respondents with accurate intervention recall, who stated they had fully stopped, or heavily decreased, their saiga horn usage following the intervention. Sum contrasts have been applied, in which "each coefficient compares the corresponding level of the factor to the average of the other levels" [1].

Did the 2019 target audience change their behaviour more than the non-target audience?

Yes - Significant

Decrease saiga horn usage = target-audience + Chinese dialect + education + generation Singaporean + religion + income

((variable levels with less than 10 participants for the target audience were omitted so they did not disproportionately skew results: 'Education Unknown', 'Religion Unknown', 'Religion Other', 'Dialect Unknown'))

Coefficients:	Estimate	Standard Error	Z-Value	Pr(>|z|)	
(Intercept)	-7.407	153.798	-0.048	0.962	
Target Audience	1.186	0.347	3.421	0.001	***
Dialect Cantonese	2.749	153.798	0.018	0.986	
Dialect Hainanese	2.255	153.799	0.015	0.988	
Dialect Hakkas	3.036	153.798	0.020	0.984	
Dialect Hokkiens	2.197	153.798	0.014	0.989	
Dialect Teochews	2.542	153.798	0.017	0.987	
Dialect Other	-12.778	768.988	-0.017	0.987	
Education Primary School (and Under)	-0.474	0.573	-0.827	0.408	
Education Secondary School / ITE	0.250	0.312	0.801	0.423	
Education Pre-University / Post-Secondary School	-0.122	0.347	-0.351	0.725	
Education University / Graduate School	0.346	0.315	1.098	0.272	
Generation First	-1.301	0.837	-1.554	0.120	
Generation Second	0.213	0.392	0.543	0.587	
Generation Third	0.687	0.351	1.960	0.050	.
Generation More than Third	-0.326	0.635	-0.513	0.608	
Generation Unknown	0.727	0.645	1.128	0.259	
Religion Buddhist	-0.064	0.359	-0.178	0.859	
Religion Catholic	-0.657	0.834	-0.788	0.431	
Religion Christian	-0.162	0.431	-0.376	0.707	
Religion Taoist	0.026	0.627	0.042	0.966	
Religion None	0.857	0.342	2.508	0.012	*
Income One	0.040	0.344	0.117	0.907	
Income Two	0.028	0.326	0.086	0.932	
Income Three	-0.136	0.538	-0.252	0.801	
Income Four	0.927	0.552	1.680	0.093	.
Income Five	-0.383	0.633	-0.605	0.545	
Income Unknown	-0.477	0.471	-1.013	0.311	

Signif. codes: 0 '***' 0.001 '**' 0.01 '*' 0.05 '.' 0.1 ' ' 1

Null deviance: 368.76 on 2004 degrees of freedom

Residual deviance: 327.51 on 1982 degrees of freedom

AIC: 373.51<br>Number of Fisher Scoring iterations: 18

Table 13. GLM output for profiles of respondents (within the target audience) with accurate intervention recall, who stated they had fully stopped, or heavily decreased, their saiga horn usage following the intervention. Sum contrasts have been applied, in which "each coefficient compares the corresponding level of the factor to the average of the other levels" [1].

Did 2019 high-fidelity users in the target audience change their behaviour more than others in the target audience?					
Yes - Significant					
**Decrease saiga horn usage = high-fidelity saiga user + Chinese dialect + education + generation Singaporean + religion + income**<br>((variable levels with less than 10 participants for the target audience were omitted so they did not disproportionately skew results: 'Education Unknown', 'Religion Unknown', 'Religion Other', 'Dialect Unknown'))					
Coefficients:	Estimate	Standard Error	Z-Value	Pr(>|z|)	
(Intercept)	-7.328	155.313	-0.047	0.962	
High-Fidelity Saiga User	1.113	0.359	3.106	0.002	**
Dialect Cantonese	2.706	155.313	0.017	0.986	
Dialect Hainanese	2.392	155.314	0.015	0.988	
Dialect Hakkas	2.902	155.313	0.019	0.985	
Dialect Hokkiens	2.158	155.313	0.014	0.989	
Dialect Teochews	2.553	155.313	0.016	0.987	
Dialect Other	-12.712	776.562	-0.016	0.987	
Education Primary School (and Under)	-0.580	0.574	-1.011	0.312	
Education Secondary School / ITE	0.314	0.310	1.013	0.311	
Education Pre-University / Post-Secondary School	-0.102	0.347	-0.295	0.768	
Education University / Graduate School	0.368	0.319	1.155	0.248	
Generation First	-1.181	0.838	-1.409	0.159	
Generation Second	0.257	0.394	0.652	0.514	
Generation Third	0.741	0.350	2.115	0.034	*
Generation More than Third	-0.461	0.642	-0.718	0.473	
Generation Unknown	0.644	0.643	1.001	0.317	
Income One	0.001	0.343	0.003	0.997	
Income Two	-0.008	0.327	-0.024	0.981	
Income Three	-0.100	0.541	-0.185	0.853	
Income Four	1.040	0.552	1.882	0.060	.
Income Five	-0.456	0.634	-0.720	0.472	
Income Unknown	-0.477	0.471	-1.012	0.312	
Religion Buddhist	-0.113	0.360	-0.315	0.753	
Religion Catholic	-0.650	0.834	-0.779	0.436	
Religion Christian	-0.053	0.428	-0.124	0.901	
Religion Taoist	-0.039	0.627	-0.063	0.950	
Religion None	0.855	0.342	2.499	0.012	*

```
---
Signif. codes:  0 ‘***’ 0.001 ‘**’ 0.01 ‘*’ 0.05 ‘.’ 0.1 ‘ ’ 1
Null deviance: 368.76  on 2004  degrees of freedom
Residual deviance: 329.76  on 1982  degrees of freedom
AIC: 375.76
Number of Fisher Scoring iterations: 18
```

Reasons for Behaviour Change

When asked why respondents did or did not decrease their saiga horn usage following the intervention, respondents with accurate intervention recall who changed their behaviour were significantly more likely to mention reasons related to the intervention message (i.e. ‘Saiga as an Animal’), whereas those who did not change their behaviour mentioned the effectiveness of saiga horn the most often (Table 14). Statistical analyses shown below.

Table 14. Reasons given for changing or not changing behaviour. Among all respondents who decreased their saiga horn usage (38 people) and among all respondents who did not decrease their saiga horn usage (22 people)

Did decrease saiga horn usage	
Saiga as an Animal (e.g. endangered species)	29
Respondent prefers alternatives	9
Saiga horn not effective	4
Saiga horn effective	2
No longer need saiga horn	1
Taste of saiga horn is undesirable	1
Don't use it often	1
Tradition	1
Did not decrease saiga horn usage	
Saiga horn effective	10
Don't use it often	5
Not big impact/Don’t Care	4
Tradition	2
Respondent prefers alternatives	2
Reason Unknown	1
Saiga horn is expensive	1

2-sample test for equality of proportions with continuity correction

Those who decreased saiga horn usage

“Saiga as an Animal” vs “Respondent prefers alternatives”

data: c(29, 9) out of c(38, 38)

X-squared = 19, df = 1, p-value = 6.536e-06

Those who did not decrease saiga horn usage

“Saiga horn effective" vs “Tradition”

data: c(10, 5) out of c(22, 22)

X-squared = 1.6184, df = 1, p-value = 0.1017

References

1. Fox J An R and S-Plus companion to applied regression. Thousand Oaks, USA.; London, UK: Sage Publications; 2002.
