# Supplementary material for: Evaluating a large-scale online behaviour change intervention aimed at wildlife product consumers in Singapore
Source: PLoS One. 2021 Mar 24;16(3):e0248144. doi: 10.1371/journal.pone.0248144 (PMC7990170; doi:10.1371/journal.pone.0248144)
Supplement: S5 File — (PDF) [file pone.0248144.s005.pdf]

S5 File: Supplementary Shopkeeper Survey Results

Table of Contents

SHOPKEEPER AND SHOP DEMOGRAPHICS **2**

CUSTOMER TREATMENT PREFERENCES **3**

PERCEPTIONS OF WILD ANIMALS' CONSERVATION STATUS **4**

INTERVENTION-SPECIFIC ANALYSES **5**

ACCURATE INTERVENTION RECALL 5
SOURCES OF EXPOSURE 5
PERCEIVED SALES CHANGES 6

Shopkeeper and Shop Demographics

Out of shopkeepers who completed the survey: 51% were female and 49% were male. Shopkeepers 35-59 years old composed 51%, followed by 18-34 year olds (27%), and 60+ year olds (23%). Shopkeepers were predominately first- and second-generation Singaporean (both at 38%). Shopkeepers who completed the survey came from chain and family-owned TCM shops the most often (Fig1). Additionally, the Planning Areas (i.e. neighbourhood districts) of Bedok, Jurong West, and Tampines yielded the largest number of completed surveys. To protect the anonymity of our shopkeepers, their Planning Area location has been withheld.

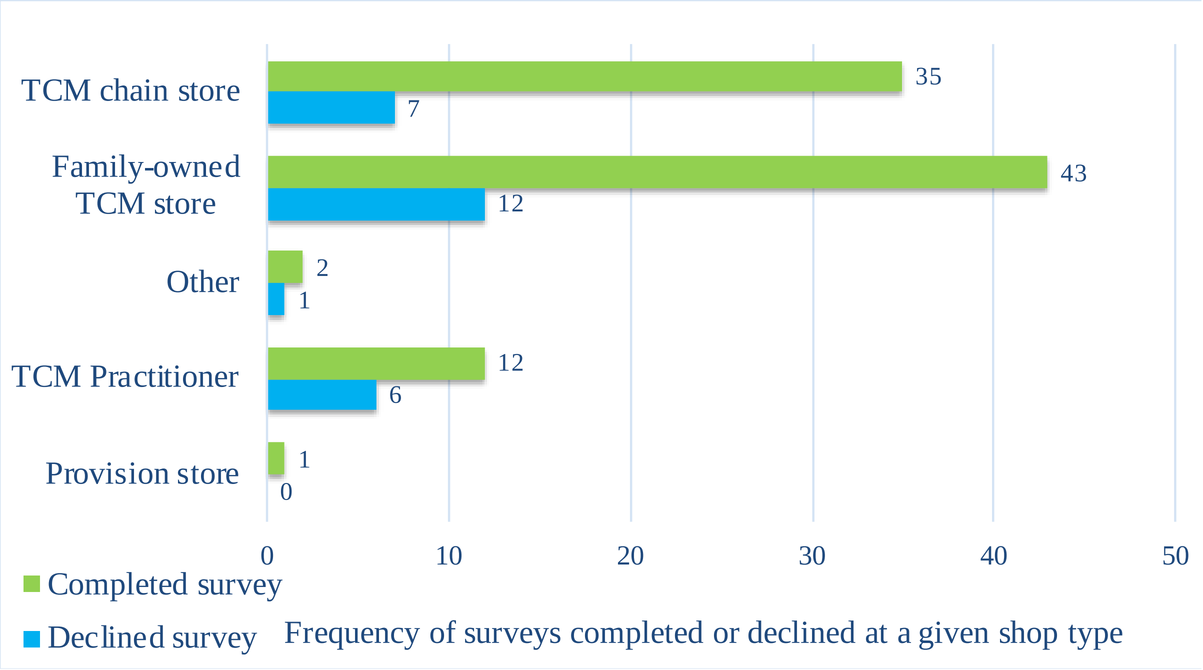

Figure 1. Frequencies of completed surveys broken down by shop type.

Customer Treatment Preferences

When asked what heatiness/fever treatments that a shopkeeper's customers preferred to purchase the most, shopkeepers stated differences in the perceived preferences of their overall customer base and their target audience (middle-aged female) specific customer base (Fig 2).

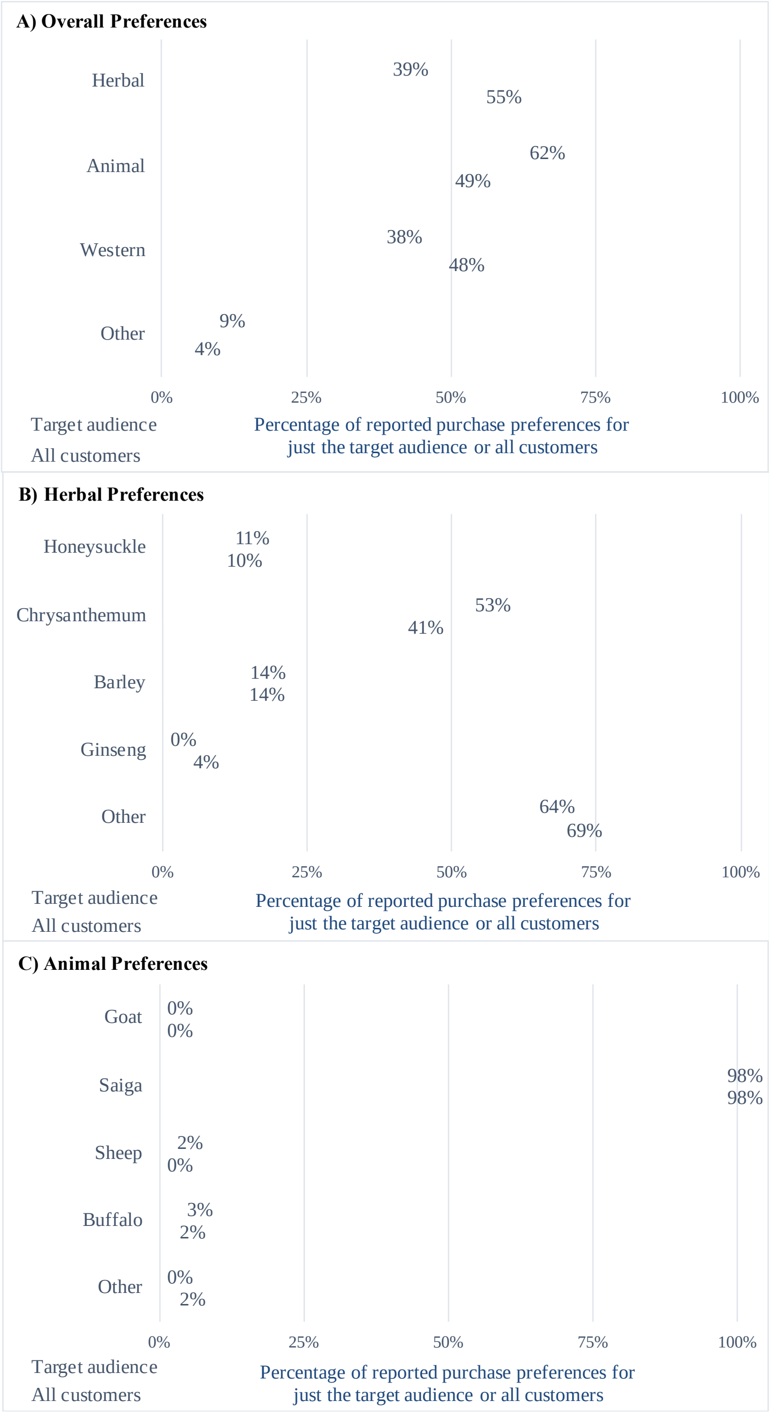

Figure 2. Trends in shopkeeper perceived customer preferences for heatiness and fever treatments. **A)** Overall customer preferences. **B)** Herbal treatment preferences. **C)** Animal treatment preferences. Percentages out of responses given by 93 shopkeepers for their target audience (middle-aged female) customer base, and their overall customer base. Preference selections were not mutually exclusive.

Perceptions of Wild Animals' Conservation Status

Shopkeepers were asked which animals (out of a list of animals used often in TCM) were common in the wild (Table 1). Sea cucumbers were most often mentioned as common in the wild. Turtles were never mentioned as common in the wild. Shopkeepers were also asked to elaborate on their answers if they wanted. The content of their responses was then coded (Table 2).

Table 1: Wild Animals perceived as common in the wild. Each animal's frequencies and percentages are shown out of total shopkeeper respondents (93 people).

Sea cucumber (hai shen)	42	(45%)
Goat (shan yang)	32	(34%)
Turtle (hai gui)	0	(0%)
Saiga antelope (ling yang)	15	(16%)
Sea horse (hai ma)	13	(14%)
Rhino (xi niu)	1	(1%)
None -- N/A in 2017	21	(23%)
I don't know	18	(19%)

Table 2: Content stated by respondents when discussing which of the given animals used in TCM a respondent thought were common in the wild. Frequencies out of total 93 shopkeepers' responses. Content were not mutually exclusive.

Saigas being protected/endangered	16
Other species' populations are doing OK	16
Saigas are restricted/Saiga's restrictions	14
Other species' farming	13
Saiga populations are decreasing	11
Saiga horn alternatives	11
Saiga horn stockpile	11
Saiga horn cost	11
Other species' populations are decreasing	9
Saiga populations are doing OK	9
Saiga hunting	8
Respondent stated they are 'not informed'	7
Saiga farming	7
Other species' protections	6
Others species' restrictions	6
Saiga horn reports from the government	3
Saiga horn imports are decreasing	2
Saigas are not killed for their horn	2
Saiga horn supply is decreasing	1
Other species' product supplies are decreasing	1
Other species' product imports are decreasing	1
ACRES non-profit stickers in TCM shopkeeper windows	1
Shopkeeper indicated they were uncomfortable to discuss details	2
Shopkeeper did not elaborate	33

Intervention-Specific Analyses

Accurate Intervention Recall

Shopkeepers mentioned a number of topics when describing what they thought the “recent media attention about ling yang” was about (Table 3). Eight shopkeepers gave a generally accurate description of the intervention message and thus were labelled as having ‘accurate recall.’

Table 3: Stated intervention recall and the content of the intervention described by the shopkeepers. ‘Saiga endangered’ indicated a generally accurate description of the intervention message. Respondents could describe more than one type of content.

Those who stated intervention recall	23
Content described by shopkeepers:	
Saiga horn is effective	10
Saiga is endangered (accurate recall)	8
Saiga horn bans/restrictions	7
Saiga horn supplies are decreasing	2
Saiga horn is not effective	2
Saiga horn stockpiles	2
Saiga horn is losing popularity	2

Sources of Exposure

Shopkeepers were asked where they heard about the “recent media attention on ling yang” (Table 4). Those with accurate and inaccurate intervention recall mentioned the news most often. Given some of the 'other' sources though, such as “company training books”, or “personal experience”, it is clear that not all shopkeepers were talking about the intervention. Some of the 'other' descriptions could be accurate though, such as "google search" or "radio broadcast".

Table 4: Sources where shopkeepers heard about the intervention message. Frequencies are given for number of shopkeepers indicating a given source. Sources were not mutually exclusive.

	**Inaccurate Recall**	**Accurate Recall**
News outlet/newspaper	9	4
Other	6	2
Social media	4	2
TCM association	3	0
Family	1	1
Friends	0	1
Colleague	0	0
I don't know	0	0

Perceived Sales Changes

When asked whether sales for saiga horn had changed at all following the recent media attention around ling yang, five shopkeepers (one shopkeeper with accurate intervention recall) stated there had been a decrease in saiga horn sales (Table 5). Shopkeepers also described why they thought saiga horn sales had decreased or stayed the same (Table 5). No shopkeepers stated saiga horn had increased in sales.

Table 5: Perceived change (if any) in saiga sales following the intervention - encompassing sales to any customer, including the target audience. Reasons given by shopkeepers for these sales trends is also shown.

Perceived decrease in sales ^	5
No perceived change in sales	16
I don't know	2
**Discussion content for perceived change/no change**	
**Decreased saiga horn sales**	
Cost of saiga	3
Prefer alternatives	2
Doctor more convenient	1
Saiga losing popularity	1
Restrictions on saiga access	1
**No change in saiga horn sales**	
Effective	11
Customer choice	3
Tradition	3
Cost of saiga	2
Doctor more convenient	2
Impact apathy	2
Prefer alternatives	2
Sockpile decreasing	1

^ About 5% of shopkeepers perceived a decrease in saiga sales, and this sales change ranged from unknown, to 25%, to >70%.
